# Chronic low back pain as a control failure state: a predictive-rhythm model of motor control impairment and functional rigidity with functional system constraint modulation

**DOI:** 10.3389/fpain.2026.1812201

**Published:** 2026-07-23

**Authors:** SeongHyung Cho, DongYeob Baek, MiSuk Jeong, MinHee Kim

**Affiliations:** 365 Madi Joint & Spine Pain Center, Busan, Republic of Korea

**Keywords:** adaptive variability, autonomic flexibility, chronic low back pain, control failure state, functional rigidity, motor control impairment, phase transition, predictive coding

## Abstract

Motor dysfunction in chronic low back pain (CLBP) is commonly described at the output level—altered movement patterns, reduced variability, and maladaptive behaviors. Yet such output-level descriptions leave recurring clinical questions unresolved: why altered patterns begin, why apparent correction often fails to generalize, why stress and physiological state modulate function, and why performance fluctuates despite stable structure. We hypothesize that a subset of CLBP presentations can be conceptualized as a persistent control failure state when stabilization-dominant regulation, reduced adaptive variability, impaired phase-transition dynamics, and constrained predictive updating become self-maintaining features of motor behavior. In this framework, stabilization does not mean trunk stability alone; it denotes a regulatory strategy that minimizes uncertainty by reducing variability, restricting phase transitions, and prioritizing predictability over adaptability. Functional rigidity is treated as the clinical output expression of this state, characterized by reduced rhythmic variability, autonomic inflexibility, and anticipatory co-contraction, with altered kinematics emerging as a downstream biomechanical expression rather than the primary explanatory level. Respiratory–autonomic dynamics are introduced not as a privileged cause of CLBP but as one clinically accessible example of a broader class of phase-transition interfaces through which constrained regulation can be observed, perturbed, and quantified during task transitions and repetition. Functional System Constraint modulation (FSCm) is therefore not proposed as a universal CLBP theory or a proprietary intervention protocol. Rather, it is a constraint-level validation architecture that aligns three interacting mechanistic features—restricted phase transitions, reduced adaptive variability, and constrained predictive updating—with measurable recovery targets: restoration of phase-transition capacity, re-expansion of adaptive variability, re-enablement of predictive updating, and re-coupling of interacting rhythms. The model yields falsifiable temporal predictions: phase-transition and autonomic changes should precede or co-occur with reductions in stabilization-based motor output, while variability and repetitive-task performance may partially dissociate from pain intensity. By positioning FSCm as a complementary framework that integrates predictive coding, motor control impairment, nociplastic pain, and behavioral models, this hypothesis specifies target populations, boundary conditions, operational markers, and temporal-order falsification criteria for mechanistic CLBP research.

## 1 Introduction

Chronic low back pain is a leading contributor to disability worldwide, and most presentations are non-specific, without an identifiable pathoanatomical cause (1, 2). Degenerative changes seen on spine imaging are also prevalent in asymptomatic populations, so observable dysfunction often reflects system-level adaptation rather than a single structural lesion (3).

Motor control changes in low back pain are heterogeneous; “tight” and “loose” control patterns can both emerge as protective adaptations, with potential long-term costs (4–6). The present hypothesis focuses on when such adaptations become persistently self-maintaining—i.e., a control failure state—rather than on any single movement pattern.

Chronic low back pain (CLBP) commonly presents with motor dysfunction (altered patterns, reduced variability, abnormal muscle activation, and avoidance) that correlates with disability and relapse risk. Yet these features are often described at the output level, leaving the underlying control state under-specified—a gap this paper addresses by formalizing motor dysfunction as a persistent control failure state.

In this paper, we propose that a subset of CLBP presentations may be conceptualized as a control failure state when stabilization-dominant regulation, reduced adaptive variability, and impaired phase-transition dynamics become persistent features of motor behavior. By “control failure,” we do not mean that the motor system fails to produce movement. Rather, we refer to a condition in which prediction updating and coordination processes become constrained such that the system cannot return to adaptive regulation and remains biased toward low-variability stabilization. Within a predictive regulation framework, ongoing pain-related perturbations and uncertainty are hypothesized to weaken flexible prediction updating and alter the precision assigned to sensory evidence. As unresolved prediction error accumulates, the system reorganizes toward a strategy that minimizes uncertainty through stabilization, expressing itself through rigidity-like motor output regimes.

A central claim of this manuscript is that this control failure state has an identifiable temporal signature: it is hypothesized to be associated with predictive–rhythmic desynchronization across coupled physiological rhythms that normally permit transitions between stability and movement. Rather than treating rhythm as a downstream consequence of pain, we treat rhythmic coordination as control architecture, linking autonomic regulation, phase-transition dynamics (including respiration), and central pattern generator (CPG)–based motor rhythms across multiple time scales (7, 8). When phase transitions are constrained, variability narrows, reduced autonomic flexibility can persist, and motor output reorganizes around co-contraction and fixation. From a pain perspective, such stabilization regimes may be compatible with sustained nociceptive salience; however, the present manuscript focuses primarily on motor-control impairment and its measurable control-state signatures.

Importantly, the present framework is not intended as a universal explanation for all chronic low back pain presentations. The framework is most applicable to CLBP patients who demonstrate a control failure signature: persistent stabilization-dominant motor strategies, reduced adaptive variability across repeated tasks, impaired phase-transition dynamics, disproportionate motor dysfunction relative to structural pathology, and failure of symptom resolution despite conventional biomechanical correction.

The present framework is not intended to replace predictive coding, Cognitive Functional Therapy (CFT), motor control impairment models, or nociplastic pain concepts. Rather, Functional System Constraint modulation (FSCm) is proposed as a complementary framework that seeks to explain how disturbances identified by these approaches may become temporally coupled, stabilized, and resistant to updating within a persistent control failure state.

Structural pathology and biomechanical loading remain important contributors to CLBP. The present framework focuses on how such perturbations become incorporated into persistent regulatory states. FSCm therefore does not replace biomechanical explanations, but seeks to explain why certain biomechanical adaptations become stabilized and resistant to updating over time.

Throughout this manuscript, respiration is treated as a practical observational window into phase-transition dynamics rather than as a primary causal mechanism. The framework remains agnostic regarding which physiological or behavioral rhythm may ultimately prove most informative. Respiration is emphasized because it offers measurable phase structure and established coupling with autonomic regulation, making it particularly suitable for hypothesis testing.

However, a theoretical model is clinically meaningful only if it yields a modifiable and measurable target. For that reason, we introduce FSCm as a translation framework—a constraint-level formalization of predictive regulation—that specifies candidate constraints expected to change as control recovers. FSCm is defined here as a technique-agnostic, constraint-level approach: it conceptualizes persistent motor control impairment as being maintained by a constraint landscape that suppresses adaptive variability, limits phase transitions, and constrains prediction updating across coupled rhythms. In this view, recovery is not achieved by correcting movement outputs alone, but by modulating the constraints that hold the system in a stabilization regime—for example, by restoring phase-transition capacity through accessible markers such as respiration, improving autonomic flexibility, re-expanding rhythmic variability, and re-enabling prediction updating within motor control.

The contribution of the present work lies not in proposing a new biological mechanism, but in operationally integrating established predictive and motor-control principles into a constraint-level framework that yields specific, testable temporal predictions. In doing so, the framework distinguishes movement output from control state, links persistence to multi-scale rhythmic desynchronization and phase-transition constraints, and uses FSCm as a technique-agnostic translation layer connecting mechanism to measurable markers and falsifiable ordering.

In this manuscript, a control failure state refers to a persistent stabilization regime in which the motor system can still produce movement but cannot readily restore adaptive variability and flexible regulation once uncertainty and perturbation are introduced. Predictive regulation denotes control in which motor behavior is organized by continuously updating predictions and weighting sensory evidence to minimize future error under uncertainty. Predictive–rhythmic desynchronization refers to a loss of flexible phase relationships across coupled autonomic, respiratory, and motor rhythmic processes that normally enable transitions between stabilization and movement generation. Finally, the constraint landscape describes the set of interacting physiological and behavioral constraints that determine whether phase transitions and variability are permitted or suppressed, thereby maintaining—or releasing—the control failure state.

For conceptual clarity, the model is organized hierarchically. The core concept is the control failure state. Its three interacting mechanistic features are restricted phase transitions, reduced adaptive variability, and constrained predictive updating. Functional rigidity, stabilization-dominant motor behavior, and motor control impairment are treated as clinical or output-level expressions of this state. Precision locking is treated as one candidate form of constrained predictive updating; autonomic inflexibility and phase fixation are operational examples nested within the mechanistic features rather than additional independent theories.

The present framework is organized around four mechanistic questions that conventional output-centered accounts do not fully resolve.

First, why does a particular movement pattern emerge in the first place? We propose that such patterns arise when the control system shifts from movement-based regulation to stabilization-dominant regulation due to constrained variability and impaired phase-transition capacity.

Second, why does correction often fail despite repeated attempts? We hypothesize that the stabilization regime becomes the system's default state, such that adaptive variability cannot be restored even when external perturbation decreases.

Third, why do stress, respiration, and emotional state modulate symptom severity? We propose that intermediate-scale autonomic–respiratory dynamics act as a transition gate linking predictive priors to motor rhythmic stability, amplifying or releasing stabilization depending on phase-transition capacity.

**Figure 1:**
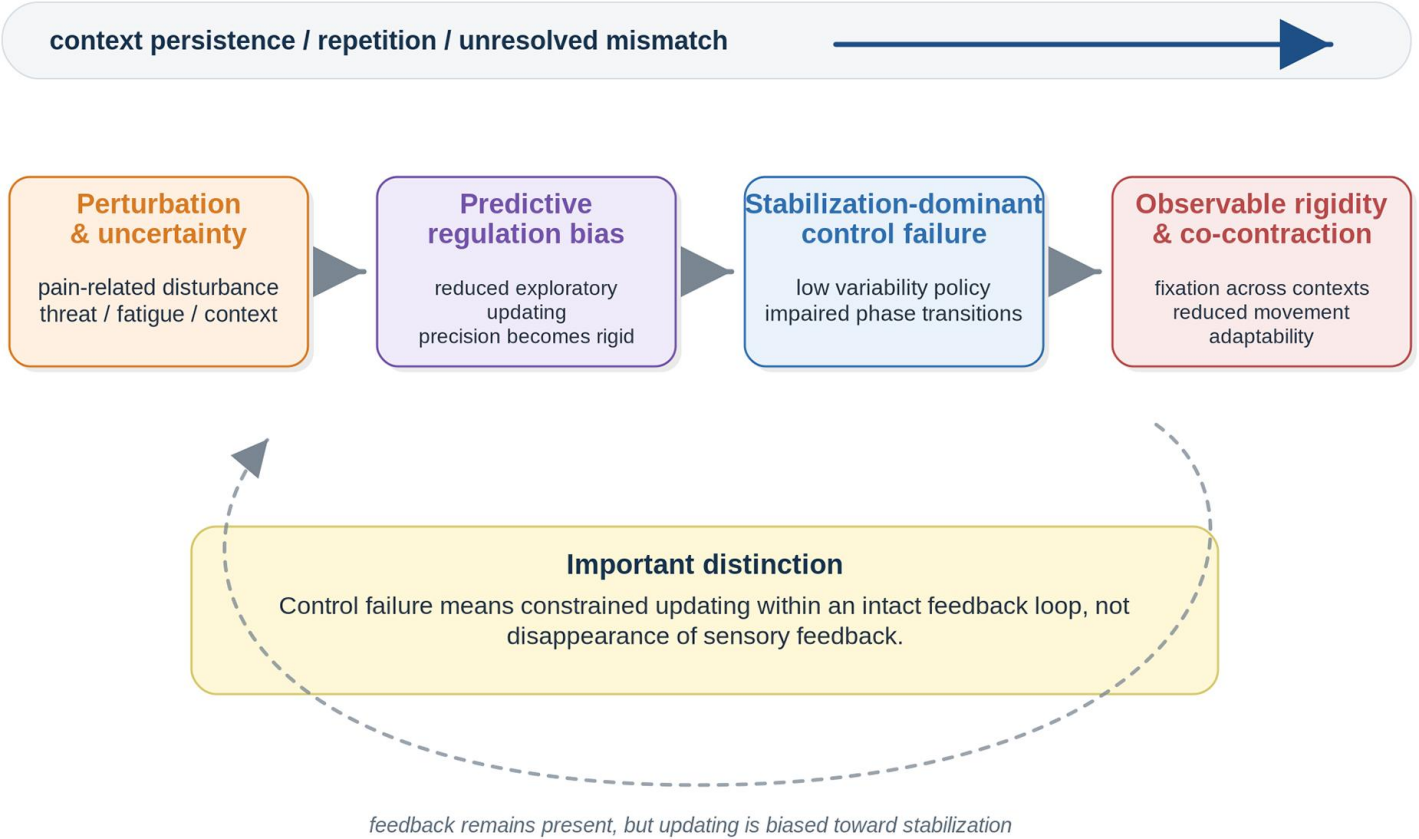
State-cascade schematic of CLBP motor control impairment. This figure intentionally summarizes the macroscopic state transition (perturbation/uncertainty → predictive regulation bias → stabilization-dominant control failure → observable rigidity/co-contraction) rather than the full closed-loop predictive architecture. The closed-loop model—including sensory feedback, prediction error, and precision modulation—is detailed in Figure 2. Control failure here denotes constrained updating (e.g., precision locking) within an intact loop, not a loss of feedback.

Fourth, why is performance inconsistent across days or contexts? We argue that CLBP reflects state-dependent dynamics rather than fixed movement defects; fluctuations reflect transient shifts in phase-transition availability and variability bandwidth rather than structural change. These four questions and the proposed pathway are summarized in Figure 1.

Accordingly, this manuscript formalizes CLBP-related motor control impairment as a control failure state defined by persistent stabilization under repetition and transition demands, and it specifies measurable state markers and falsifiable temporal predictions to evaluate this claim.

This paper does not propose a new anatomical substrate, a novel pain mechanism, or a specific therapeutic protocol. Instead, it formalizes a system-level control failure condition and specifies how such a condition can be operationalized, tested, and potentially reversed through constraint modulation.

The novelty of this manuscript lies not in introducing a new biological substrate, but in formalizing a time-sequenced control-state model with an operational definition, a minimal marker panel, and explicit falsification criteria. Specifically, we predict a testable lead–lag ordering whereby phase-transition interface dynamics (operationalized through the selected interface, such as respiratory phase alternation, trunk–pelvis/gait-related phase variability, or repeated-task phase markers depending on context) and autonomic flexibility change before—or co-occur with—reductions in stabilization-based motor output and re-expansion of adaptive variability.

**Figure 2:**
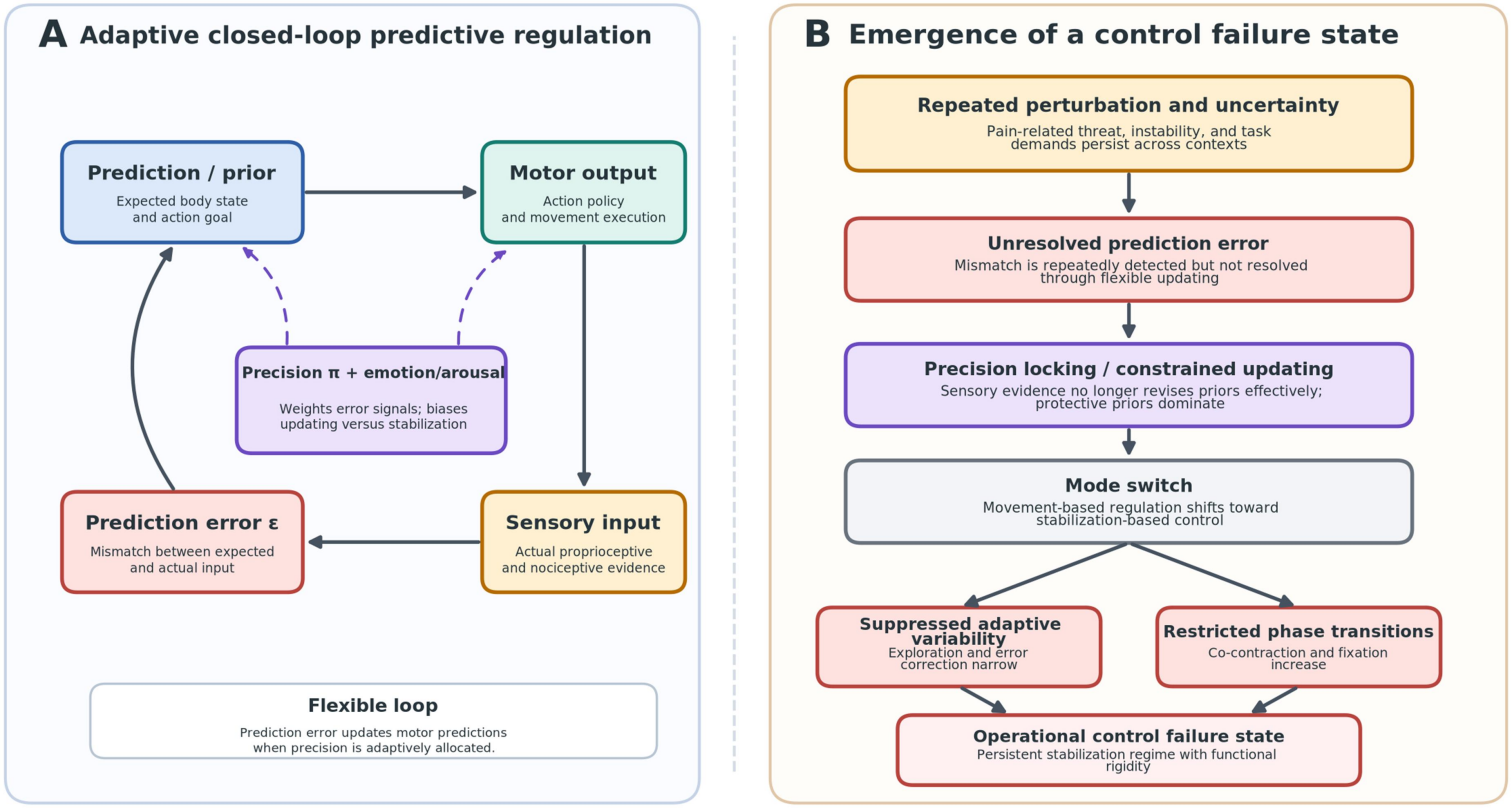
Adaptive closed-loop predictive regulation and emergence of a control failure state. **(A)** Flexible predictive regulation loop consisting of prediction/prior, motor output, sensory input, and prediction error, modulated by precision and emotion/arousal, leading to adaptive updating. **(B)** Progression from repeated perturbation and uncertainty to unresolved prediction error, constrained updating, and a mode switch resulting in suppressed adaptive variability, restricted phase transitions, and an operational control failure state characterized by functional rigidity.

What this framework adds beyond existing models is not a new biological component, but an explicit, testable specification of a failure domain: (i) CLBP motor control impairment is defined as a time-sequenced control failure state (distinct from output-level movement descriptions), (ii) the state is operationalized with markers that emerge under repetition/transition demands and a minimal measurement panel, and (iii) explicit temporal-order predictions provide falsification criteria. Figures 1, 2 provide schematic overviews of the conceptual pathway and closed-loop architecture, and Table 1 summarizes the core construct distinctions used throughout the manuscript.

**Table 1:** Movement deficit, motor control impairment, control failure state, and functional rigidity in CLBP: four-question alignment and falsifiable distinctions.

Construct	Core definition	4Q link	Key markers	Falsification criterion
Movement Deficit	Observable deviation in movement outcome (e.g., ROM, coordination, speed)	Q1: pattern emergence (descriptive; does not specify mechanism)	ROM, kinematic deviation, task accuracy	If output correction alone restores adaptability across repetition and removes fluctuation independent of autonomic/respiratory change, control failure is not required.
Motor Control Impairment	Reduced adaptability of regulation across contexts (impaired prediction updating, variability modulation)	Q2: correction fails to generalize	Variability modulation under perturbation; context-dependent instability	If adaptability normalizes through strength/output correction without restoring phase-transition capacity, control-level impairment may not be primary.
Control Failure State	Persistent stabilization-dominant regulatory state with suppressed adaptive variability and impaired phase transitions, persisting under repetition/transition demands (not defined by a single movement phenotype).	Q1–Q4: convergence via persistence + phase-transition failure	EMG co-contraction; respiratory phase-transition index; HRV flexibility; repetition-based phase fixation	If stabilization persists while phase-transition capacity and variability remain intact, or if pain fully explains marker change, the model is weakened.
Functional Rigidity (Output Regime)	State-dependent motor output regime characterized by anticipatory (pre-movement) co-contraction and phase fixation under repetition/uncertainty (distinct from passive stiffness or ROM limitation).	Q3–Q4: stress modulation + contextual fluctuation as output regime	Pre-movement EMG; respiratory–motor coupling; reduced rhythmic variability	If rigidity markers do not co-vary with autonomic/respiratory dynamics, rhythmic interface claims are unsupported.

A detailed glossary, additional scope notes, constraint classes, validation options, boundary examples, and biomechanical expression-layer details are provided in Supplementary Texts S0–S4.

## 2 Motor control impairment vs. movement deficit

Motor dysfunction in CLBP is often framed as a problem of movement—a deviation from an expected kinematic pattern, a reduction in range of motion, or an observable coordination “fault.” While these observations are clinically salient, they primarily describe output error: what the movement looks like relative to a normative reference. Output error, however, is not equivalent to the state of the control system that generated the output. A similar movement pattern may be produced by qualitatively different control strategies (e.g., flexible regulation vs. rigid stabilization), and a visibly “abnormal” pattern may be an adaptive response under uncertainty rather than a primary deficit. For this reason, a mechanistic account of CLBP requires a distinction between movement deficit as an observable phenomenon and motor control impairment as a disturbance of regulation.

In this manuscript, we define movement deficit as a measurable deviation in movement outcome or capacity, including changes in range, speed, accuracy, or coordination, typically assessed through kinematics, clinical observation, or task performance. Movement deficits are often context-dependent and may fluctuate with pain, threat, fatigue, or environmental demands. Importantly, they do not specify whether the nervous system is able to update predictions and restore variability when conditions change. In contrast, motor control impairment refers to a disruption in the processes that enable adaptive movement regulation, such as prediction updating, sensorimotor integration, and the dynamic allocation of stability vs. mobility. Motor control impairment is therefore not defined by a single movement pattern; it is defined by reduced adaptability across contexts, particularly when uncertainty or perturbation requires flexible reorganization.

This distinction becomes clearer when the motor system's regulatory objective is considered. Output-centered models implicitly treat the goal of control as improving movement accuracy or reducing deviation from an ideal trajectory. In a predictive regulation framework, however, the primary objective is not accuracy *per se*, but predictive stability under uncertainty, achieved through the controlled management of variability. Variability is not simply noise to be eliminated; it is a functional degree of freedom that enables exploration, error correction, and phase transitions between movement generation and stabilization. When prediction updating is intact, variability can expand or contract as needed. When prediction updating becomes constrained, the system may suppress variability to preserve short-term stability, even at the cost of long-term adaptability.

This is the point at which control failure state becomes conceptually necessary. We use this term to denote a persistent condition in which the motor system remains locked into a stabilization-dominant regime and cannot readily restore adaptive variability and flexible regulation once uncertainty and perturbation are introduced. In such a state, the system may produce movement that appears acceptable in simple tasks, yet fails when demands require repeated phase transitions, multi-joint coordination, or tolerance of sensory ambiguity. Control failure is therefore characterized less by the magnitude of output error and more by the persistence of low-variability stabilization strategies, including co-contraction, phase fixation, and reduced rhythmic adaptability.

Importantly, the control failure state is not a synonym for guarding, stiffening, avoidance, or a “tight control” strategy as a descriptive phenotype. Those labels describe observable behaviors; control failure state denotes persistence of stabilization-dominant regulation under repetition/transition demands and failure to re-open phase transitions even when task demands change or pain fluctuates. In this sense, Control failure (state) → functional rigidity (output regime).

These distinctions also clarify why conventional clinical endpoints—particularly pain intensity and range of motion—are often insufficient to characterize motor dysfunction in CLBP. Pain ratings primarily reflect perceptual and affective processing rather than the organization of motor control, and they can decrease without resolving stabilization lock. Range of motion measures capacity under a constrained test context, but they do not capture whether the system can maintain rhythmic variability, update predictions, or transition between stability and movement during repeated or uncertain conditions. Thus, range of motion (ROM) and pain can improve while the control system remains in a failure state, leaving functional rigidity, reduced adaptability, and relapse risk largely unaddressed.

Accordingly, assessment should differentiate (i) output-level deficits (what movement is produced), (ii) control-level impairment (how regulation adapts across contexts), and (iii) persistent control failure states (when stabilization dominates despite changing demands). This framework motivates the use of markers that index control persistence and rhythmic adaptability, such as variability measures, co-contraction patterns, phase transitions in respiration-linked movement tasks, and objective indicators of phase fixation during repetitive actions. By formalizing these distinctions, the model shifts the focus from correcting movement outputs to identifying and modulating the control constraints that maintain maladaptive stabilization. Put differently, output correction is not equivalent to control-state recovery. If phase transitions remain restricted under repetition or uncertainty, the system may revert toward stabilization even after apparent improvement in single-trial output. In this framing: Control failure (state) → functional rigidity (output regime). Altered kinematics are downstream expressions. These construct-level distinctions are summarized in Table 1.

### 2.1 Relationship to existing CLBP models

Predictive coding provides an important account of why pain-related expectations may persist. FSCm extends this perspective by focusing on the conditions under which prediction updating becomes progressively constrained and stabilization-dominant regulation emerges.

Cognitive Functional Therapy (CFT) has clarified how beliefs, threat, protective behavior, and lifestyle context shape disabling CLBP. FSCm does not challenge these behavioral insights; it seeks to complement them by specifying physiological and motor-regulatory mechanisms through which protective behaviors may become persistent and resistant to change.

Existing motor control impairment models describe altered movement patterns and coordination strategies, including stabilization-dominant (“tight control”) and movement-variable (“loose control”) adaptations. FSCm focuses on the subset of presentations in which stabilization-dominant strategies become self-maintaining, low-variability regimes rather than flexible context-dependent adaptations.

Finally, FSCm is compatible with nociplastic pain concepts and may provide a complementary framework for understanding how persistent pain-related alterations become expressed through motor and autonomic regulation. Its intended contribution is therefore integrative rather than competitive: it connects predictive, behavioral, motor, and nociplastic processes through the concept of persistent control failure.

## 3 Predictive regulation framework: an operational definition of the control failure state

A central aim of this manuscript is to move beyond output descriptions of motor dysfunction in CLBP and specify a control-level mechanism that can be evaluated and tested. We therefore formalize motor control impairment as a consequence of predictive regulation failure, in which the nervous system becomes progressively constrained in its capacity to update motor predictions and flexibly allocate precision to sensory evidence. Within this framework, persistent perturbation (pain-related disturbance, uncertainty, and threat-weighted interpretations) drives a transition from movement-based regulation to stabilization-based control. The defining feature of this transition is not simply increased “stiffness,” but a shift in how the system handles prediction error over time.

### 3.1 Predictive regulation as closed-loop control under uncertainty

Predictive regulation assumes that motor behavior is controlled by minimizing expected future error rather than by reacting to present error alone. In practice, this involves a continuous cycle: the system generates predictions about sensory consequences of action, compares predicted and actual sensory input, updates the prediction model based on the resulting error, and selects subsequent actions accordingly. Crucially, the system also needs to estimate how much to trust different information streams—i.e., it needs to assign precision to sensory evidence and priors, which determines the influence of incoming feedback vs. internal predictions (9). This closed-loop predictive control cycle is schematized in Figure 2.

In CLBP, persistent perturbation and uncertainty may progressively bias this precision allocation. As pain becomes coupled with threat and instability, the system may treat sensory discrepancy as high-cost, reducing exploratory variability and favoring conservative stabilization outputs. This creates a setting in which prediction error is not used to update flexible motor predictions, but instead triggers protective regulatory shifts that suppress variability (10).

### 3.2 From prediction error accumulation to mode switching

Within this framework, prediction error accumulation is a time-dependent process. It does not mean that error becomes arbitrarily large at every moment. Rather, it denotes a failure to resolve error through adaptive updating, leading to a gradual increase in persistent mismatch across repeated actions and contexts. When unresolved error accumulates, a mode switch becomes likely: the system shifts away from movement generation and toward stabilization. This shift can be understood as a control-level strategy to reduce uncertainty by restricting degrees of freedom, increasing co-contraction, and narrowing the range of allowable phase transitions in rhythmic control.

One candidate form of constrained predictive updating is precision locking: a state in which precision is rigidly allocated such that incoming sensory evidence no longer effectively updates predictions, or conversely, predictions are no longer flexibly revised in response to evidence. Either form of locking reduces adaptive learning and promotes control persistence. Over time, precision locking may yield a characteristic signature: reduced variability, impaired phase transitions, and a stabilization regime that becomes self-maintaining even when perturbation decreases.

### 3.3 Operational definition of the control failure state

To make the theory testable, we define the control failure state operationally. The control failure state is not equivalent to pain severity, a specific movement pattern, or structural findings. It is defined by the persistence of a stabilization-dominant control regime and by measurable signatures that indicate constrained predictive updating and constrained rhythmic flexibility.

This construct is intentionally upstream of output labels (e.g., guarding or avoidance): it is defined by persistence and transition failure under repetition/uncertainty demands, not by a particular movement phenotype or by pain intensity alone.

Operational definition (control failure state): A control failure state is present when stabilization becomes the system's default regulatory mode prior to overt movement deviation and, under uncertainty or perturbation (e.g., repeated tasks, contextual threat, sensory ambiguity), the motor system exhibits (i) suppressed adaptive variability, (ii) impaired phase transitions across coupled rhythms, and (iii) stabilization output persistence—including pre-movement anticipatory co-contraction and fixation strategies—that fails to normalize despite changing task demands or improvements in pain intensity.

This operational definition implies several measurable consequences. First, variability is not merely low; it is inflexible, failing to expand when exploration or adaptation is required. Second, phase transitions—particularly those linked to respiratory and autonomic modulation—are diminished or delayed, resulting in persistent reduced autonomic flexibility and low rhythmic variability. Third, stabilization outputs can persist across contexts, producing reliable markers such as pre-movement electromyography (EMG) co-contraction, co-contraction dominance, and phase fixation during repetitive tasks.

Thus, the defining feature is not a specific movement pattern, but persistence of stabilization under repetition/transition demands that normally require phase alternation and variability re-expansion.

Minimal criteria (measurement panel): To avoid circularity, the control failure state should be identified under repetition and transition demands, not from single-trial output. A minimal panel requires (i) evidence of restricted phase-transition capacity during transitions/repetition (indexed here by respiratory phase alternation or another interface marker) and (ii) reduced autonomic flexibility across the same epochs, indexed for example by heart rate variability (HRV), with optional corroboration from stabilization-output markers (e.g., anticipatory co-contraction/tonic drive) and variability/repetition-success metrics.

### 3.4 Implications for measurement and falsifiability

Because the proposed mechanism is a closed-loop failure, it generates falsifiable predictions. If the model is correct, then improvements in pain or range of motion can occur without a corresponding restoration of rhythmic variability and phase transitions. Conversely, early restoration of phase transition capacity (e.g., respiratory phase alternation and autonomic rhythmic variability) should precede or accompany improvements in motor adaptability even before pain fully resolves. These claims can be tested by tracking temporal ordering among respiratory phase dynamics, autonomic tone (e.g., HRV), motor rhythmic variability, and task-level performance across repeated-task paradigms.

Predictive constructs such as priors and precision are treated here as inferred control variables rather than directly measurable quantities in a single clinical test. Accordingly, predictive variables are operationalized indirectly through observable signatures (variability bandwidth, phase-transition capacity, and repetition-induced phase fixation), and falsification relies on temporal-order relationships among measurable markers rather than direct estimation of precision itself.

To address terminology, stabilization is defined here as a regulatory strategy rather than a synonym for desirable trunk stability. In the present framework, stabilization denotes a control policy through which the system minimizes uncertainty by reducing variability, restricting phase transitions, and prioritizing predictability over adaptability.

Whole-body stabilization refers to the extension of this strategy across interacting motor, autonomic, respiratory, and behavioral systems. Stabilization markers are operationalized as persistent co-contraction, elevated tonic drive, reduced adaptive variability, impaired phase-transition dynamics, reduced autonomic flexibility, and repetition-linked phase fixation.

**Brief glossary of core constructs** (see Supplementary Text S0.1 for detailed definitions and literature support).

Prediction: An internally generated estimate of the expected sensory consequences of action, used to guide motor output before feedback is fully available (9, 10).

Prediction error: The mismatch between predicted and actual sensory input; in predictive regulation, error is used to update predictions and refine motor control (9, 10).

Prediction error accumulation: A time-dependent increase in unresolved mismatch across repeated actions or contexts, reflecting insufficient adaptive updating rather than momentary error magnitude (9, 10).

Precision: The estimated reliability (confidence) assigned to sensory evidence or priors, determining how strongly each influences updating and control decisions (9, 10).

Predictive updating constraint: A higher-level term for conditions in which prediction error fails to support flexible learning and adaptation. Precision locking is treated as one operational example of this constraint (9, 10).

Stabilization: A regulatory strategy through which the system minimizes uncertainty by reducing variability, restricting phase transitions, and prioritizing predictability over adaptability; it is not synonymous with desirable trunk stability (4–6).

Whole-body stabilization: Extension of the stabilization strategy across interacting motor, autonomic, respiratory, and behavioral systems (4–6).

Stabilization markers: Operational markers including persistent co-contraction, elevated tonic drive, reduced adaptive variability, impaired phase-transition dynamics, reduced autonomic flexibility, and repetition-linked phase fixation (4–6).

Control failure state (operational): A persistent stabilization-dominant regime in which adaptive variability and flexible phase transitions fail to re-emerge under repetition, uncertainty, or perturbation (4, 9).

Functional rigidity: The clinical/output expression of a control failure state, characterized by anticipatory co-contraction, reduced rhythmic variability, and impaired transition capacity rather than by passive stiffness or ROM limitation (4, 5).

Concept hierarchy: Core concept = control failure state; mechanistic features = restricted phase transitions, reduced adaptive variability, constrained predictive updating; clinical expressions = functional rigidity, stabilization-dominant motor behavior, and motor control impairment (4, 9).

**Precision locking:** A stabilization-biased regulatory state in which threat-related priors are assigned excessive precision, reducing the capacity of incoming sensory evidence to update predictive models. It is inferred from reduced adaptive variability, persistent stabilization-dominant behavior, impaired proprioceptive updating, and reduced autonomic flexibility (9, 10).

**Predictive-rhythmic desynchronization:** Loss of coordinated phase relationships among predictive, autonomic, respiratory, and sensorimotor rhythms; evaluated through reductions in phase-transition capacity, adaptive variability, autonomic flexibility, and sensorimotor coordination, including HRV, trunk–pelvis coordination variability, movement stereotypy, and proprioceptive accuracy (7, 8).

**Avoidance-based stabilization:** A protective control strategy in which movement organization is biased toward minimizing anticipated threat rather than maximizing adaptability; inferred from persistent co-contraction, reduced movement variability, repetitive stabilization-dominant patterns, diminished exploratory behavior, and continued reliance on protective strategies despite reduced structural demand (5, 6).

These constructs are not proposed as independently measurable entities. In FSCm, they are higher-order regulatory constructs inferred from converging patterns across autonomic flexibility, proprioceptive performance, movement variability, trunk–pelvis coordination, and repeated-task behavior. This operational approach is intended to support empirical testing while avoiding circular definitions.

### 3.5 Biomechanical expression of control failure

In the present framework, biomechanical findings are not interpreted as primary causal mechanisms of chronic low back pain, but rather as observable expressions of a persistent control failure state. FSCm does not reject structural pathology or biomechanical loading models (1–5). Instead, it seeks to explain how perturbations arising from structural, sensory, or pain-related factors become stabilized within a regulatory system and subsequently manifest as recurrent biomechanical patterns.

When phase-transition capacity is reduced, adaptive variability becomes constrained and predictive updating becomes less effective. Under these conditions, the motor system increasingly adopts stabilization-dominant strategies designed to minimize uncertainty rather than optimize movement adaptability. The consequence is not merely altered movement, but a characteristic biomechanical expression of persistent control failure.

One important manifestation is altered trunk–pelvis coordination. Under normal conditions, trunk and pelvic segments continuously redistribute loading through flexible phase relationships that adapt to changing task demands. Within a control failure state, however, trunk–pelvis interactions become increasingly rigid and stereotyped, reflecting reduced adaptability rather than efficient movement regulation. As coordinative flexibility decreases, loading redistribution becomes progressively constrained, increasing reliance on a narrower repertoire of movement solutions. This reduction in coordinative flexibility may contribute to recurrent movement strategies and difficulty adapting to environmental or task-related perturbations.

A second manifestation is spinal loading adaptation. FSCm proposes that chronic low back pain is not solely associated with increased spinal loading, but with reduced flexibility in how loading is distributed and redistributed across movement contexts. Persistent stabilization strategies may result in repeated loading concentration patterns, increasing exposure to mechanical stress while simultaneously reducing exploratory movement behavior. In this view, loading concentration is not interpreted as an isolated mechanical defect, but as a biomechanical expression of stabilization-dominant regulation.

A third manifestation is reduced movement variability. Adaptive variability normally allows the motor system to explore alternative movement solutions, update predictions, and maintain robustness under uncertainty. In a control failure state, variability becomes progressively restricted, resulting in repetitive and predictable movement solutions that prioritize stability over adaptability. This reduction in movement variability is hypothesized to contribute to persistent motor control impairment and functional rigidity.

Importantly, the magnitude and expression of these biomechanical manifestations may vary across CLBP presentations. Some individuals may primarily exhibit stabilization-dominant trunk–pelvis coordination patterns and loading concentration, whereas others may demonstrate relatively preserved movement variability but persistent phase-transition constraints or autonomic inflexibility. FSCm therefore focuses on shared control failure signatures rather than assuming a single biomechanical phenotype across all CLBP presentations. Collectively, altered trunk–pelvis coordination, spinal loading adaptation, and movement variability represent biomechanical expression layers through which the underlying control failure state becomes observable in clinical practice. These biomechanical manifestations are therefore viewed not as independent causes of chronic low back pain, but as measurable outputs of a stabilization-dominant regulatory regime. These biomechanical expressions provide one of the primary pathways through which the control failure state becomes clinically observable in CLBP. The relationship between control failure, biomechanical expression, and clinical presentation is summarized schematically later in the manuscript. Additional biomechanical expression details are provided in Supplementary Text S4.

### 3.6 Target population and boundary conditions

FSCm is not intended to explain all presentations of CLBP. The framework is most applicable to patients who demonstrate a control failure signature: persistent stabilization-dominant motor strategies; reduced adaptive variability across repeated tasks; impaired phase-transition dynamics; disproportionate motor dysfunction relative to structural pathology; and failure of symptom resolution despite conventional biomechanical correction.

The framework is not primarily intended to explain acute nociceptive pain, progressive neurological disorders, severe structural instability, tumor, infection, fracture-related pain, or other red-flag spinal conditions. These presentations may require different diagnostic and therapeutic priorities, although control failure processes may coexist secondarily in some cases.

## 4 Rhythmic organization of motor control: rhythm as control architecture

Motor control is commonly discussed in terms of what is produced—muscle activation patterns, joint kinematics, or coordinated sequences. Here, rhythm is treated not as a downstream pattern but as the temporal architecture that gates sensory sampling, prediction updating, and stability–mobility switching under uncertainty. When this rhythmic scaffolding degrades, stabilization can become the default, contributing to both motor rigidity and pain persistence.

Accordingly, we fix a central claim of the present model as follows:

Rhythmic breakdown is proposed as a time-domain signature of control failure.

When rhythmic coordination deteriorates, the system loses the capacity to execute phase transitions, regulate variability, and revise predictions across repeated actions. What emerges clinically as rigidity, co-contraction, or phase fixation is therefore interpreted as a time-domain signature of a control system that has shifted into stabilization-dominant regulation.

### 4.1 Rhythm as a temporal solution to control under uncertainty

Predictive regulation requires integration across time: predictions are generated, errors computed, precision allocated, and updating occurs within appropriate windows. Rhythmic organization provides these windows by structuring control into phase-dependent cycles that coordinate sensory sampling and motor output. Adaptive control therefore depends not only on what is predicted, but on whether rhythmic timing reliably permits error resolution and phase transitions between stability and movement.

This perspective clarifies why reducing variability can be maladaptive. Variability is not simply noise but a control resource for exploration, error correction, and adaptability. Rhythmic integrity allows variability to be flexibly modulated; rhythmic breakdown collapses this flexibility and promotes stabilization as a default regime.

### 4.2 Multi-timescale coupling: fast, intermediate, and slow dynamics

We propose that motor control in CLBP is organized by the coupling of rhythmic processes operating across at least three interacting time scales:

Fast scale (sensorimotor/CPG dynamics):

At the fastest scale, motor control relies on rapid sensorimotor loops and CPG-related rhythmic organization to coordinate movement timing, multi-joint sequencing, and task-specific phase relationships. This scale supports moment-to-moment coordination and is highly sensitive to perturbations that disrupt timing, phase locking, and rhythmic variability.

In CLBP, these fast-scale motor rhythms become clinically visible through trunk–pelvis coordination, gait-related variability, repeated sit-to-stand performance, and lifting-related movement organization. These biomechanical expressions provide observable manifestations of how control failure becomes embedded within everyday movement behavior.

Intermediate scale (autonomic and affective modulation): At an intermediate scale, autonomic and affective processes shape the gain and flexibility of motor control through arousal-dependent modulation of variability, muscle tone, and respiratory phase dynamics. This layer influences whether the system tolerates exploration or shifts toward conservative stabilization. Persistent reduced autonomic flexibility and reduced respiratory phase alternation represent intermediate-scale constraints that can propagate downward to fast motor rhythms (11, 12).

Slow scale (predictive priors and long-term regulation): At the slowest scale, predictive priors encode longer-term expectations about threat, stability, and control costs. These priors shape how uncertainty is interpreted and how precision is assigned over time. When slow-scale priors become biased toward threat and instability, the system becomes more likely to suppress variability and lock into stabilization, even in contexts where movement-based regulation would otherwise be possible.

**Figure 3:**
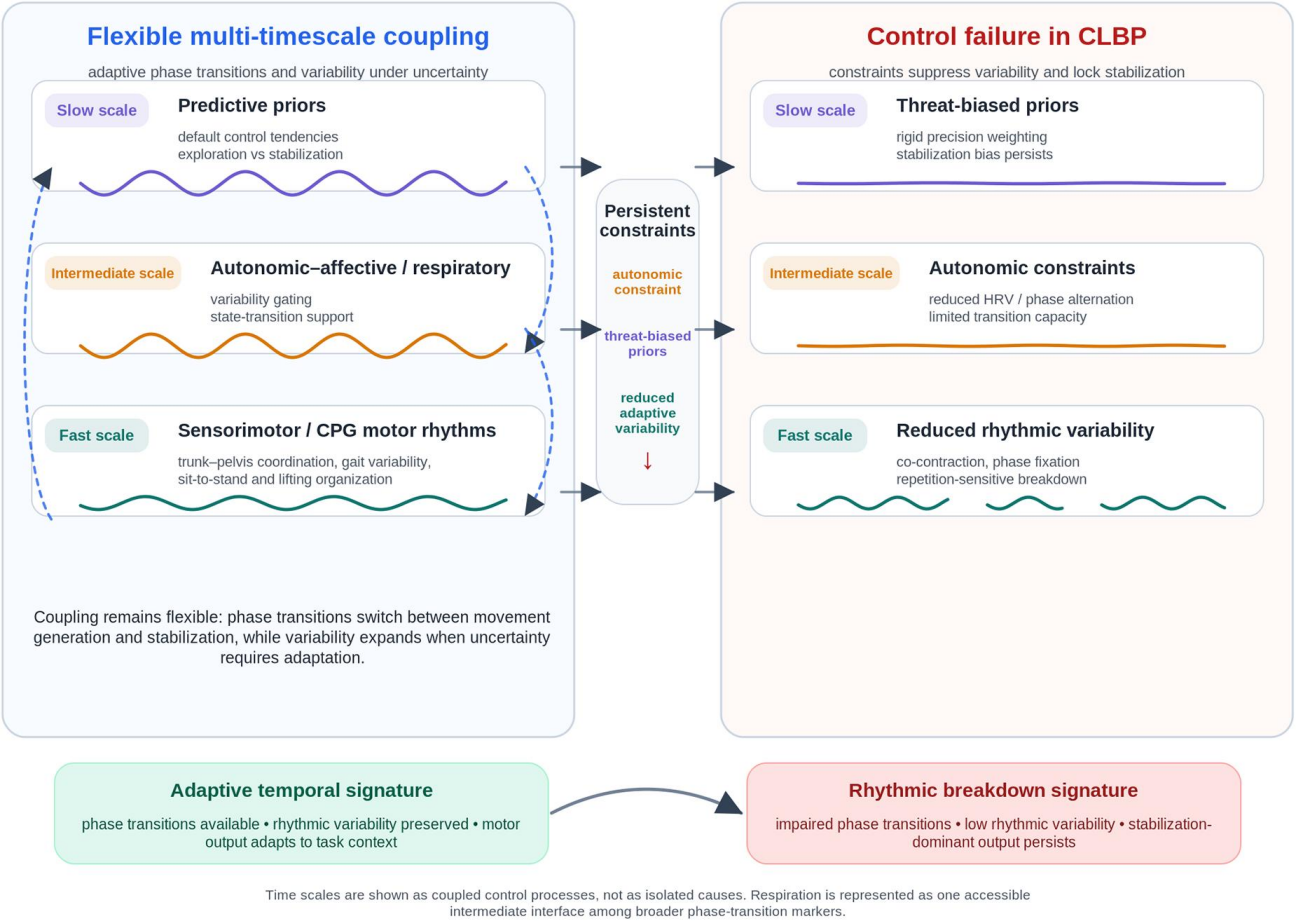
Multi-timescale coupling in motor control and the temporal signature of control failure. Motor control is organized by coupled rhythmic processes operating across fast (sensorimotor/CPG), intermediate (autonomic–affective and respiratory), and slow (predictive priors) time scales. Fast-scale motor rhythms may be clinically expressed through trunk–pelvis coordination, gait-related variability, repeated sit-to-stand performance, and lifting-related movement organization. Flexible coupling enables phase transitions between movement generation and stabilization while regulating variability under uncertainty. In CLBP, persistent autonomic constraints and threat-biased priors suppress fast rhythmic variability and impair phase transitions, producing rhythmic breakdown as a proposed time-domain signature of a control failure state.

These time scales are not independent. Healthy regulation depends on coupling with flexible phase transitions, allowing shifts between stabilization and movement generation as demands change. Control failure in CLBP emerges when coupling becomes rigid: intermediate autonomic constraints reduce fast-scale variability and phase flexibility, while slow-scale threat-biased priors bias precision allocation so stabilization persists rather than remaining context-specific. This multi-timescale coupling framework is illustrated in Figure 3.

### 4.3 Rhythmic breakdown as a pathway to functional rigidity

Within this multi-timescale framework, functional rigidity is not treated as a direct response to pain intensity alone. Instead, it is viewed as the result of a progressive loss of rhythmic flexibility across coupled systems. As intermediate and slow dynamics constrain variability, the fast sensorimotor system increasingly relies on co-contraction and phase fixation to achieve short-term stability. This produces a recognizable signature: reduced variability in kinematics, reduced alternation in respiration-linked phases, reduced HRV and autonomic flexibility, and reduced adaptability during repetitive tasks. Candidate rhythm domains and measurable markers are summarized in Table 2.

**Table 2:** Rhythm domains across time scales and candidate measurable markers in CLBP.

Time scale	Rhythm domain	Functional role in control	Candidate measurable markers	Expected change in control failure
Fast	Sensorimotor/CPG-related motor rhythms	Phase coordination, sequencing, task timing, variability for adaptation	EMG timing/phase indices, co-contraction metrics; kinematics variability (stride-to-stride, trunk–pelvis coordination); task repetition stability	Reduced kinematic variability; increased co-contraction; phase fixation during repetitive tasks
Intermediate	Autonomic–respiratory rhythms	Variability gating, tonus regulation, phase transition support	HRV (time/frequency/nonlinear metrics); respiratory phase alternation/transition measures; respiratory–cardiac coupling indices	Reduced HRV; impaired respiratory phase alternation; reduced autonomic flexibility
Intermediate	Affective/arousal modulation rhythms (optional/contextual)	Precision modulation, threat weighting, tolerance of uncertainty	Optional: state measures of arousal; physiological arousal markers; coupling with respiratory/HRV markers	Optional/contextual: increased arousal persistence; reduced flexibility in state transitions
Slow	Predictive priors (longer-term control tendencies)	Sets default precision tendencies; biases toward stabilization vs. exploration	Behavioral indices of rigidity/adaptability across contexts; longitudinal stability of variability measures; self-report constructs (as supportive context)	Increased stabilization bias; reduced adaptability across contexts; persistence despite symptom fluctuation
Cross-scale coupling	Multi-rhythm phase relationships	Enables phase transitions and adaptive regulation	Respiration–HRV coupling; respiration–motor coordination; cross-correlation/phase-locking measures	Decoupling or rigid coupling; impaired transitions; failure to recover variability after perturbation

The central implication is that rehabilitation in CLBP cannot be reduced to output correction or strengthening alone. If rhythmic architecture is compromised, output gains can appear in constrained tests while phase transitions fail under uncertainty, leaving relapse and pain persistence risk high. Rhythm may therefore need to be evaluated and targeted as a control-level property, not merely observed as a downstream pattern.

## 5 Phase-transition interfaces in CLBP: respiration as one clinically accessible example

**Figure 4:**
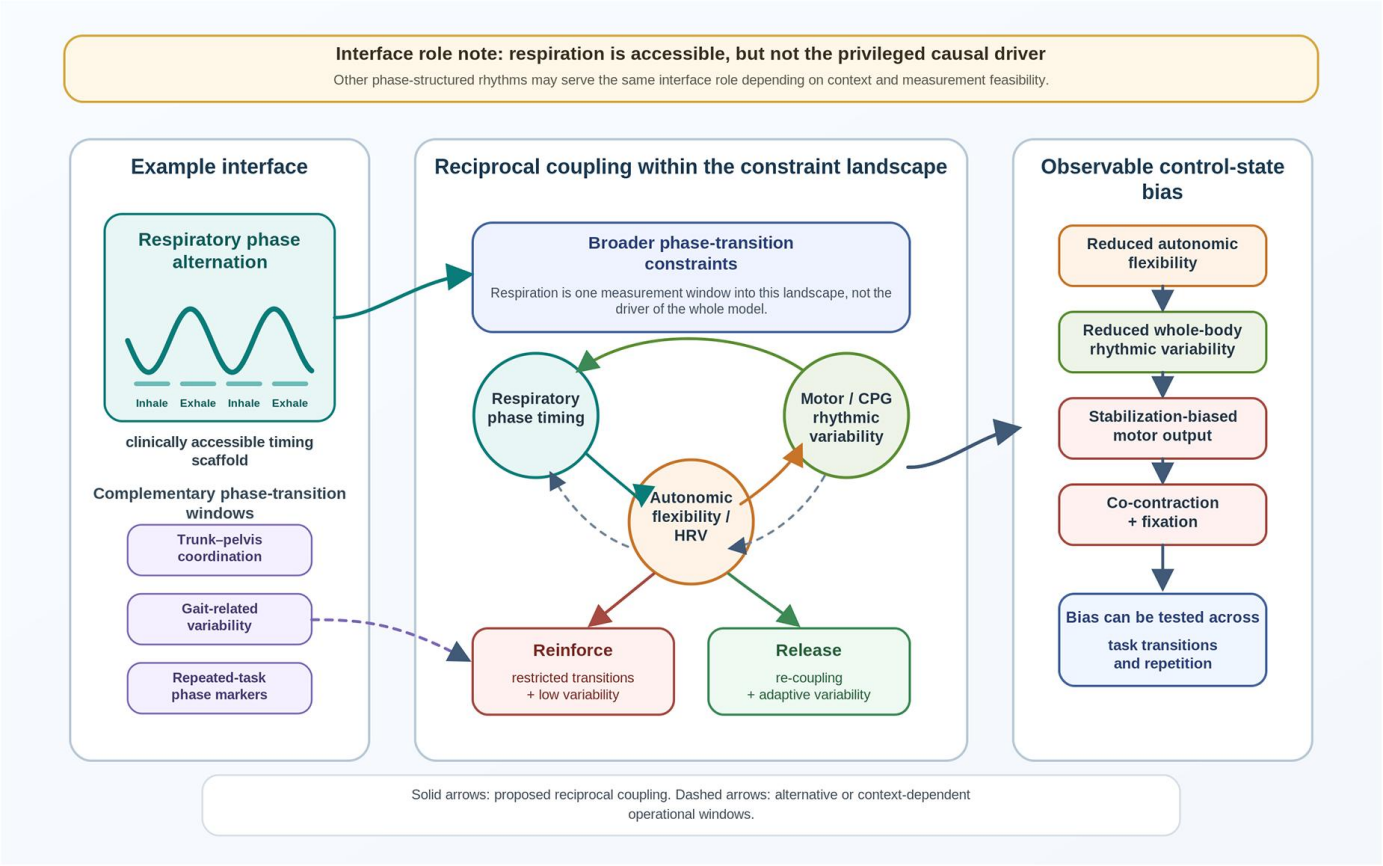
Respiratory phase alternation as an example phase-transition interface (not a privileged driver) associated with control failure in CLBP. Respiratory phase alternation provides an explicit timing scaffold that can be used as a clinically accessible measurement window into broader phase-transition constraints within the system's constraint landscape, without implying that respiration is a primary causal driver. Constrained respiratory phase transitions are proposed to co-occur with reduced autonomic flexibility and reduced whole-body rhythmic variability, biasing motor output toward stabilization-based control characterized by co-contraction and fixation. These relationships are framed as reciprocal coupling rather than a one-way causal chain, such that respiratory, autonomic, and motor rhythms can reinforce or release one another across repetition and task transitions. Other phase-structured rhythms could serve as interfaces; respiration is selected here primarily for feasibility and coupling strength.

Phase-transition failure in CLBP may become observable through multiple interfaces, including trunk–pelvis coordination, gait variability, repeated-task performance, autonomic dynamics, and respiratory phase alternation. Among these interfaces, respiration is emphasized in the present manuscript because it provides a practical and physiologically explicit measurement window that is strongly coupled to autonomic regulation and can be repeatedly assessed across tasks. Many accounts treat altered breathing in CLBP as a consequence of pain, stress, or deconditioning. Here, respiratory phase alternation is used as a repeatable timing scaffold that is bidirectionally coupled with autonomic and postural control, making it useful for assessing whether the system can switch between stabilization and movement-based regulation under repetition. Respiration is therefore not proposed as a privileged cause of motor impairment; it is one interface for detecting constrained phase transitions. This interface framing and its coupling relations are schematized in Figure 4.

### 5.1 Respiration as an interface, not a privileged cause

Respiration is not assumed to be causal. In the present framework, respiration is not privileged as a primary driver of motor control impairment in chronic low back pain (CLBP). Instead, it is introduced as a physiologically explicit candidate interface at which failures of phase transition within a stabilization-dominant control regime can be directly observed and operationalized. In principle, any rhythm with an explicit phase structure could serve as an interface; respiration is emphasized here primarily for feasibility and its strong bidirectional coupling with autonomic and postural control.

Respiration is emphasized because it has an explicit phase structure, strong bidirectional coupling with autonomic and postural control, and practical feasibility for repeated measurement across tasks—properties that make it sensitive to stabilization-dominant regulation.

Accordingly, respiration is treated here as a window into system-level control organization, not as a singular origin of dysfunction.

### 5.2 Phase transition failure and stabilization-based regulation

Adaptive motor control requires the capacity to alternate between stabilization and movement generation. Within predictive regulation, such alternation depends on tolerating variability, updating predictions, and reorganizing coordination when demands change. Phase transitions—orderly shifts between functional states—are therefore central to control recovery.

Stress and arousal are proposed to bias precision allocation toward threat-weighted priors, narrowing variability tolerance and favoring stabilization-based regulation. Because respiratory phase alternation is tightly coupled to autonomic flexibility, stress-induced reduced autonomic flexibility may restrict phase transitions, providing a clinically accessible window into the broader stabilization regime.

Respiratory rhythm provides an explicit instantiation of phase transitions. Normal inspiration–expiration alternation is tightly coupled to autonomic tone and postural control. When phase transitions are intact, respiratory timing can flexibly adjust to task demands, supporting whole-body coordination and variability regulation.

Respiratory phase transition failure refers to a condition in which this alternation becomes persistently constrained. Prolonged inspiratory dominance, reduced expiratory relaxation, and diminished respiratory variability are commonly observed under persistent arousal-related autonomic activation and uncertainty. Within this model, such patterns are interpreted as markers of a broader shift toward stabilization-based regulation, rather than isolated respiratory abnormalities (13, 14).

### 5.3 Linking respiration, autonomic tone, and motor output

The relevance of respiratory phase transition failure to motor control lies in its coupling to autonomic and musculoskeletal systems (15–17). Respiratory rhythm is bidirectionally linked to autonomic regulation: changes in respiratory timing influence vagal modulation and heart rate variability (HRV) (18–21), while autonomic state biases respiratory patterning. This coupling provides a plausible pathway through which persistent reduced autonomic flexibility may stabilize respiratory rhythm in a low-variability configuration (22, 23).

When expiratory relaxation is reduced and inspiratory bias persists, tonic stabilization tends to increase and motor output is biased toward co-contraction and fixation. This relationship is not assumed to be a one-way causal chain; rather, constrained respiratory phase dynamics, autonomic tone, and stabilization-based motor output are hypothesized to reinforce one another within a reciprocal loop.

### 5.4 Why respiratory interfaces reveal control failure under repetition

A defining feature of the proposed control failure state is its persistence under repetition. Single movements may appear adequate, yet repeated task demands reveal an inability to restore variability and phase transitions. Respiratory phase dynamics are particularly well suited to exposing this phenomenon because they provide a continuous temporal reference across repetitions and tasks.

When respiratory phase alternation remains constrained across repeated movements, the system is less able to modulate autonomic tone and redistribute muscular activity. This increases reliance on co-contraction and fixation strategies that may succeed in the short term but impose cumulative cost. Accordingly, constrained respiration can function as a sensitive indicator of repetition-induced stabilization, aligning with the broader class of repetition-based phase fixation markers discussed elsewhere in this manuscript.

### 5.5 Alternative interfaces and boundary conditions

The present framework does not claim that respiration is the only possible interface through which control failure can be detected. Other rhythms—such as autonomic oscillations, affective dynamics, or task-specific motor rhythms—may serve analogous roles depending on context, measurement feasibility, and research aims.

Respiration is emphasized here because it offers a rare convergence of explicit phase structure, multi-domain coupling, and practicality for repeated assessment. However, the core theoretical claim concerns phase transition failure and variability suppression, not the anatomical specificity of respiration. If future work shows that alternative interfaces capture these dynamics more reliably in certain contexts, the framework accommodates such substitution.

### 5.6 Testable implications

Framing respiration as one accessible interface yields falsifiable predictions without making it a required pathway. If a selected phase-transition interface reflects a stabilization-dominant control regime, then (i) restricted interface variability should co-occur with or precede reduced autonomic flexibility, (ii) constrained phase dynamics should be associated with increased muscular co-contraction under repeated task demands, and (iii) restoration of the selected interface should accompany or precede recovery of adaptive motor variability, potentially independent of pain intensity. For respiration, these predictions can be tested through respiratory phase alternation; for CLBP-specific biomechanical interfaces, trunk–pelvis coordination, gait-related variability, or repeated-task phase markers may provide more direct access to control dynamics.

Failure to observe these relationships across appropriately selected phase-transition interfaces would require revision of the proposed interface role within the predictive–rhythm model.

## 6 Functional rigidity: stabilization-dominant output regime

The term rigidity is often used as if it were a static trait or a purely psychological expression of fear and avoidance. Here, functional rigidity is defined as a reproducible, state-dependent motor output regime in which the system prioritizes stabilization over movement generation. It is not reducible to subjective distress or any single “abnormal” movement pattern; it emerges when autonomic and respiratory constraints suppress rhythmic flexibility and bias motor output toward persistent co-contraction and postural fixation (24–29).

What it is/what it is not: Functional rigidity is not passive mechanical stiffness, reduced range of motion, or a fear-avoidance behavior label. It is a state-dependent motor output regime characterized by anticipatory co-contraction, reduced phasic modulation, and repetition-sensitive phase fixation, and it is identified by persistence and context dependence rather than by a single static measure. In this manuscript, control failure state refers to the upstream regulatory condition, whereas functional rigidity refers to its observable output regime.

### 6.1 Functional rigidity as a state-dependent motor regime

A regime-based account is necessary because rigidity is not uniformly present across all contexts. Many individuals with CLBP can perform relatively well in low-uncertainty tasks but exhibit pronounced stiffening, reduced variability, and phase fixation when demands increase (e.g., repetition, perturbation, social threat, or time pressure). This suggests that rigidity is not best interpreted as a deficit of capacity, but as a control state that becomes dominant under particular regulatory conditions—especially those characterized by elevated arousal and reduced tolerance for variability.

Within the predictive regulation framework, rigidity is the motor expression of a stabilization-dominant control mode. When prediction updating is constrained and rhythmic phase transitions are impaired, the system reduces degrees of freedom to maintain short-term stability. The resulting output regime is characterized by increased tonic drive, reduced variability, and higher reliance on co-contraction patterns that “lock” segments together. This strategy can succeed in minimizing uncertainty in the moment, but it also reduces adaptability, increases metabolic cost, and can help maintain a control failure state over time. In some presentations, this stabilization bias may be detectable as anticipatory, pre-movement co-contraction before movement initiation.

### 6.2 From phase-transition restriction to co-contraction and postural fixation

A candidate pathway consistent with this model is that respiratory phase restriction indexes conditions that bias motor regime selection. When expiratory relaxation is diminished and inspiratory bias persists, opportunities for down-regulating arousal and releasing muscle tone are reduced. Because expiration is normally associated with relative parasympathetic recruitment (18, 19, 22), this shift is expected to bias the system toward more persistent sympathetic tone, elevated tonic drive, and stabilization-dominant motor output.

In this framework, co-contraction is not treated as a voluntary “guarding behavior” alone, but as an emergent control solution when phase alternation and rhythmic variability cannot be restored. Over repeated cycles, co-contraction patterns can become easier to re-enter and harder to exit, resulting in fixation at the level of trunk–pelvis coordination, other segmental coordination patterns, or segmental mobility, depending on the individual's control signature.

### 6.3 A continuous transition from movement generation to stabilization lock

**Figure 5:**
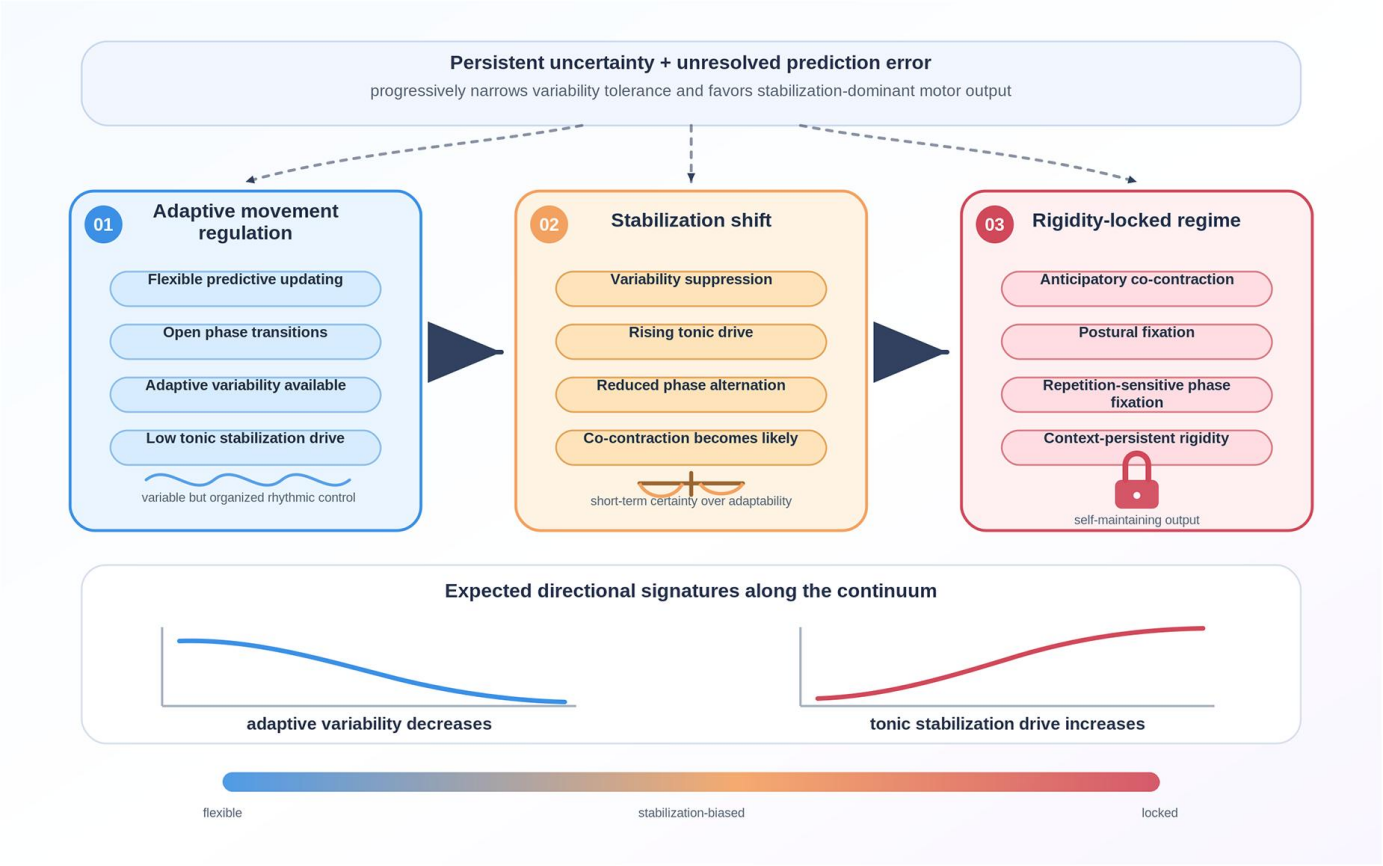
Functional rigidity as a stabilization-dominant output regime. The model conceptualizes rigidity as a continuous transition from adaptive movement regulation to a stabilization shift and ultimately to a rigidity-locked regime. Under persistent uncertainty and unresolved prediction error, adaptive variability is suppressed and tonic stabilization drive increases, biasing motor output toward co-contraction and postural fixation across contexts.

An important implication of the regime perspective is that rigidity should be conceptualized as a continuum: from adaptive movement regulation (flexible updating and phase transitions), to a stabilization shift (variability suppression and rising co-contraction), and—when persistent across contexts—to a rigidity-locked regime in which fixation becomes self-maintaining. This conceptual continuum is illustrated in Figure 5.

This continuum helps explain heterogeneity and the weak mapping between symptoms and rigidity magnitude. A patient may report relatively low pain while remaining in a stabilization regime with reduced adaptability, while another may report high pain with rigidity expressed only under certain contexts. The key distinction is whether the system can return to adaptive rhythmic control after perturbation, or whether it remains locked into stabilization output (30–33).

### 6.4 Measurement implications: distinguishing rigidity regime from pain and ROM

Because functional rigidity is an output regime, it should be identified through markers that capture state-dependence, variability suppression, and phase transition impairment, rather than through pain ratings alone. Pain intensity may covary with arousal, but it does not directly quantify the persistence of stabilization. Similarly, range-of-motion tests may demonstrate capacity but cannot reveal whether the control system can safely reintroduce variability during repeated tasks.

The model therefore motivates multi-domain measurement that includes respiratory patterning, autonomic tone, neuromuscular co-contraction, and kinematic variability. Importantly, these markers should be evaluated under conditions that reveal regime selection—especially repetition, perturbation, or uncertainty—because rigidity often manifests most clearly when the system is required to execute phase transitions rather than merely reach an endpoint. Candidate markers and operational examples are summarized in Table 3.

**Table 3:** Candidate markers of functional rigidity as an output regime in CLBP. Respiration is treated as a clinically accessible interface rather than a privileged causal driver.

Domain	What the marker captures	Example measures	Expected pattern in rigidity lock
Respiratory patterning	Phase transition/alternation capacity; expiratory relaxation (interface marker; not privileged driver)	Inspiratory bias indices; expiratory time/variability; phase transition timing	Reduced phase alternation; reduced expiratory relaxation; narrowed respiratory variability
Autonomic tone (HRV)	State flexibility; parasympathetic recruitment	Time-domain HRV; frequency-domain HRV; nonlinear HRV metrics	Reduced HRV; reduced dynamic range across tasks; reduced autonomic flexibility
EMG co-contraction	Stabilization-dominant output regime	Co-contraction indices (trunk/hip/shoulder); tonic activity level	Increased co-contraction; elevated tonic baseline; reduced phasic modulation
Kinematic variability	Availability of adaptive degrees of freedom	Stride-to-stride variability (gait cycle); trunk–pelvis coordination variability; repeated sit-to-stand or lifting performance decline; analogous non-lumbar phase-fixation marker when clinically relevant (system-level proxy; not assumed lumbar cause).	Reduced variability; phase fixation in repeated tasks; reduced adaptability under perturbation
Cross-domain coupling	Multi-rhythm coordination integrity	Respiration–HRV coupling; respiration–movement phase coupling	Rigid or weakened coupling; impaired phase transitions across domains

## 7 Repetitive movement failure and avoidance-based stabilization: avoidance as automated control output

Repetitive movement is a fundamental requirement of daily function and a strong test of motor control integrity. In CLBP, impairments often become most visible not during single-trial performance but during repetition, when the system needs to repeatedly execute phase transitions, maintain variability, and update predictions in the face of accumulating uncertainty and sensory discrepancy. This section proposes that a characteristic feature of the control failure state is repetitive movement failure, expressed as progressive loss of rhythmic flexibility and increasing reliance on stabilization strategies across repetitions.

Critically, the model reframes “avoidance” in mechanistic terms. Avoidance is often treated as a psychological label describing fear-driven behavior. In the present framework, avoidance-based stabilization is conceptualized as an automated control output that emerges when predictive updating and rhythmic phase transitions remain constrained. In other words, avoidance is not the cause of control failure; it is a phenotypic expression of a motor system that has shifted into stabilization-dominant regulation.

### 7.1 Repetition as a control stress test

Single movements can often be completed through compensations that mask underlying control limitations. Repetition, by contrast, increases control demands in three ways. First, repeated actions amplify unresolved prediction error when sensory discrepancies persist across trials. Second, repetition requires reliable phase transitions (e.g., alternation between stabilization and mobility) and exposes phase transition failure. Third, repetition demands variability modulation: successful repetition requires the system to allow small fluctuations that prevent fatigue and preserve adaptability, rather than collapsing into a rigid solution (34–39).

For these reasons, repetitive tasks are well suited to operationalize control failure. The key phenomenon is not merely that performance declines, but that the system increasingly selects stabilization-based outputs—co-contraction, reduced variability, and fixation—over flexible movement generation.

### 7.2 Phase fixation as an objective marker of repetitive failure

The framework proposes that repetitive failure in CLBP can be indexed by objective signs of phase fixation, reflecting constrained coordination under repetition. These markers are intended as system-level proxies of stabilization-dominant regulation under repetition, not as lumbar causal mechanisms. Candidate examples should prioritize lumbar-relevant repeated demands, including reduced trunk–pelvis coordination variability, reduced gait-cycle variability, or performance decline during repeated sit-to-stand or lifting tasks. Analogous non-lumbar repeated-task phase-fixation markers may be considered when clinically relevant. When the system operates under stabilization lock, repeated actions may show progressively reduced phase variability, increased tonic recruitment, and diminished adaptability even if single-trial range of motion appears acceptable.

The purpose of these markers is not to claim that any single anatomical site is the origin of CLBP. Rather, they are introduced as measurable proxies for the persistence of a stabilization regime and the inability to restore variability during repetition. In this model, any non-lumbar phase-fixation marker is only one instance of a broader class of repetitive-task phase fixation signatures that may appear primarily in trunk–pelvis coordination, gait, sit-to-stand, lifting, or other repeated movements depending on the individual's control signature.

### 7.3 Failure learning and the automation of avoidance-based stabilization

A central mechanism proposed here is failure learning, in which repeated episodes of unsuccessful phase transitions (or perceived instability during repetition) are encoded as a high-cost prediction error signal. Over time, the system reduces the probability of entering states that previously produced error and increases the probability of selecting conservative stabilization. This process can be described as a shift from movement-based error resolution toward stabilization-based error avoidance.

Importantly, this transformation does not require explicit conscious fear to be present at every moment. Once learned, stabilization can become the default policy under uncertainty. This is why avoidance in CLBP often appears automatic and context-sensitive: the system selects co-contraction, reduced variability, and reduced movement amplitude as a precautionary output regime when conditions resemble those associated with prior failure.

Thus, “avoidance” is reframed as a control-level phenomenon: it is the behavioral expression of a regulatory policy that has become locked to minimize uncertainty, rather than a purely psychological preference to avoid pain.

### 7.4 A learned stabilization loop that reinforces control failure

**Figure 6:**
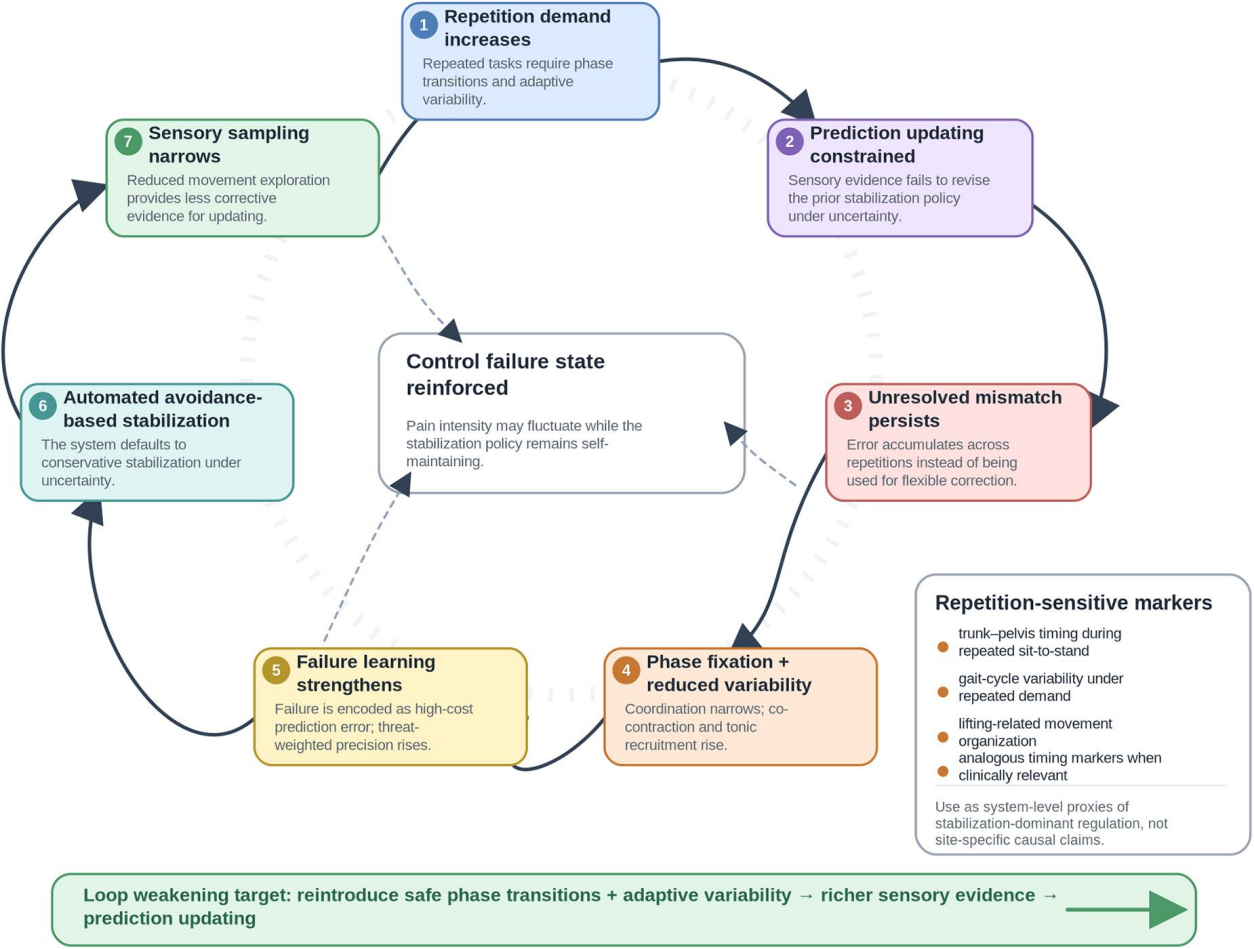
Repetitive movement failure and the automation of avoidance-based stabilization. Repetition increases demands for phase transitions and adaptive variability. When prediction updating remains constrained, unresolved mismatch persists across repetitions and promotes phase fixation and reduced variability. Repeated failure reinforces a conservative stabilization policy, which becomes automated as avoidance-based stabilization. This output regime narrows sensory sampling, further limiting prediction updating and reinforcing the control failure state. Phase fixation markers (e.g., trunk–pelvis timing during repeated sit-to-stand/gait/lifting, with analogous non-lumbar timing markers when relevant) are intended as system-level proxies of stabilization-dominant regulation under repetition, not as site-specific causal claims.

The interaction between repetitive failure and avoidance-based stabilization forms a self-reinforcing loop. Repetition increases demands for phase transitions and variability; constrained predictive updating and reduced rhythmic flexibility increase the likelihood of failure; failure increases threat-weighted precision and encourages stabilization; stabilization suppresses variability and narrows sensory sampling; and reduced sampling further limits prediction updating, making future transitions even harder. The loop can therefore reinforce the control failure state even when pain intensity fluctuates. Accordingly, days on which state transitions are more available would be expected to show better recovery of variability and fewer phase-fixation signatures during repetition, despite stable structure. This self-reinforcing repetition–avoidance loop is schematized in Figure 6.

This loop clarifies why interventions that focus exclusively on reducing pain or increasing range of motion may not restore function if they do not change the underlying regulatory policy. Recovery would be expected to involve reintroducing safe phase transitions and variability in a way that allows the system to update predictions and revise its stabilization bias.

### 7.5 Additional implications for repetition-linked validation

Partial dissociation between pain intensity and control recovery: Improvements in repetitive-task success and restoration of movement variability can occur with minimal or non-proportional changes in pain intensity, indicating partial dissociation between symptom ratings and control-level recovery.

Operationalization: track pain ratings and repetition-based performance/variability markers across sessions, and test whether control recovery predicts function/disability beyond pain intensity (e.g., mixed-effects models controlling for pain) and whether marker trajectories show lead–lag relationships that are not fully mediated by pain change.

Phase fixation as a persistence marker: Objective markers of phase fixation during repetitive tasks (e.g., reduced trunk–pelvis coordination variability during repeated gait cycles, sit-to-stand, or lifting tasks; or an analogous non-lumbar repeated-task marker when clinically relevant) will predict persistence of stabilization regimes better than single-trial kinematics or range-of-motion measures.

Operationalization: compare predictive value of repetition-based phase metrics vs. single-trial outputs for disability or relapse indicators.

Learning-loop signature: The coupling between repetitive failure (declining success across repetitions) and stabilization outputs (co-contraction and reduced variability) will strengthen over time in individuals with persistent control failure, reflecting automation of avoidance-based stabilization.

Operationalization: longitudinally assess repetition blocks; test increasing within-subject correlation between failure indices and stabilization markers.

## 8 Functional system constraint modulation (FSCm): a constraint-level translation layer for modulating control failure constraints

The preceding sections specify how motor control impairment in CLBP can persist as a control failure state through constrained predictive updating, reduced rhythmic flexibility, and stabilization-dominant output. Here, we introduce Functional System Constraint modulation (FSCm) only as a translation layer—i.e., a constraint-level formalization that clarifies candidate constraints that would be expected to change as control recovers.

The term FSCm is used solely as a shorthand label for this constraint-level framework and does not imply a proprietary system, protocol, or predefined intervention. FSCm simply denotes the constraint-level claim that persistent control failure is maintained when phase transitions, adaptive variability, and prediction updating remain restricted—and that recovery would be expected to involve modulation of these maintaining constraints. The FSCm label is optional but serves as a concise shorthand to keep mechanistic claims, markers, and modifiable constraints aligned across study design and clinical tracking.

**Figure 7:**
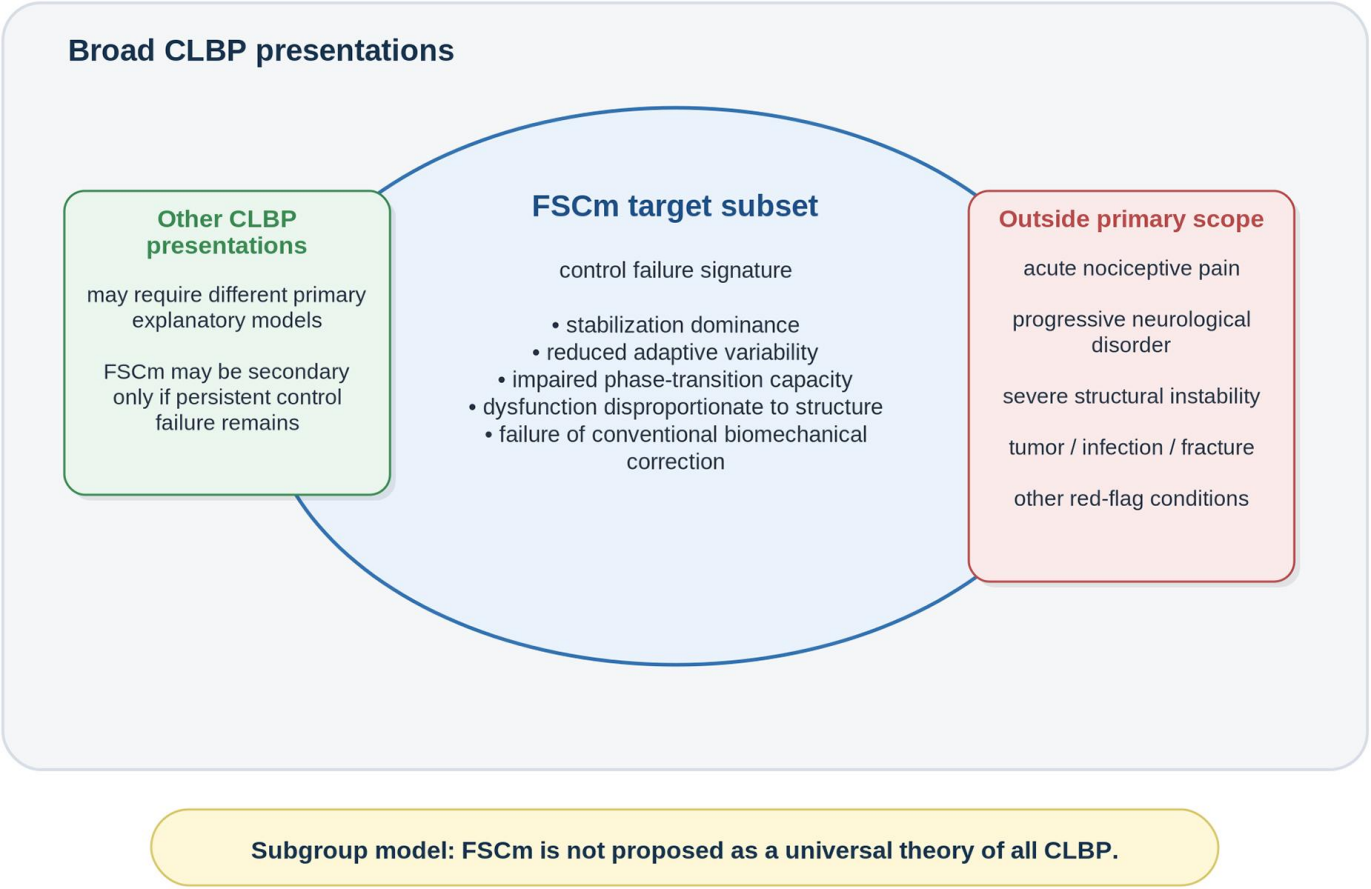
Boundary conditions of FSCm. The figure distinguishes the broad category of CLBP from the subset most applicable to FSCm: presentations showing a control failure signature with stabilization dominance, reduced adaptive variability, impaired phase-transition capacity, disproportionate motor dysfunction relative to structural pathology, and failure of conventional biomechanical correction. Acute nociceptive pain, progressive neurological disorders, severe structural instability, tumor, infection, fracture, and other red-flag conditions are shown outside the primary explanatory scope.

**Figure 8:**
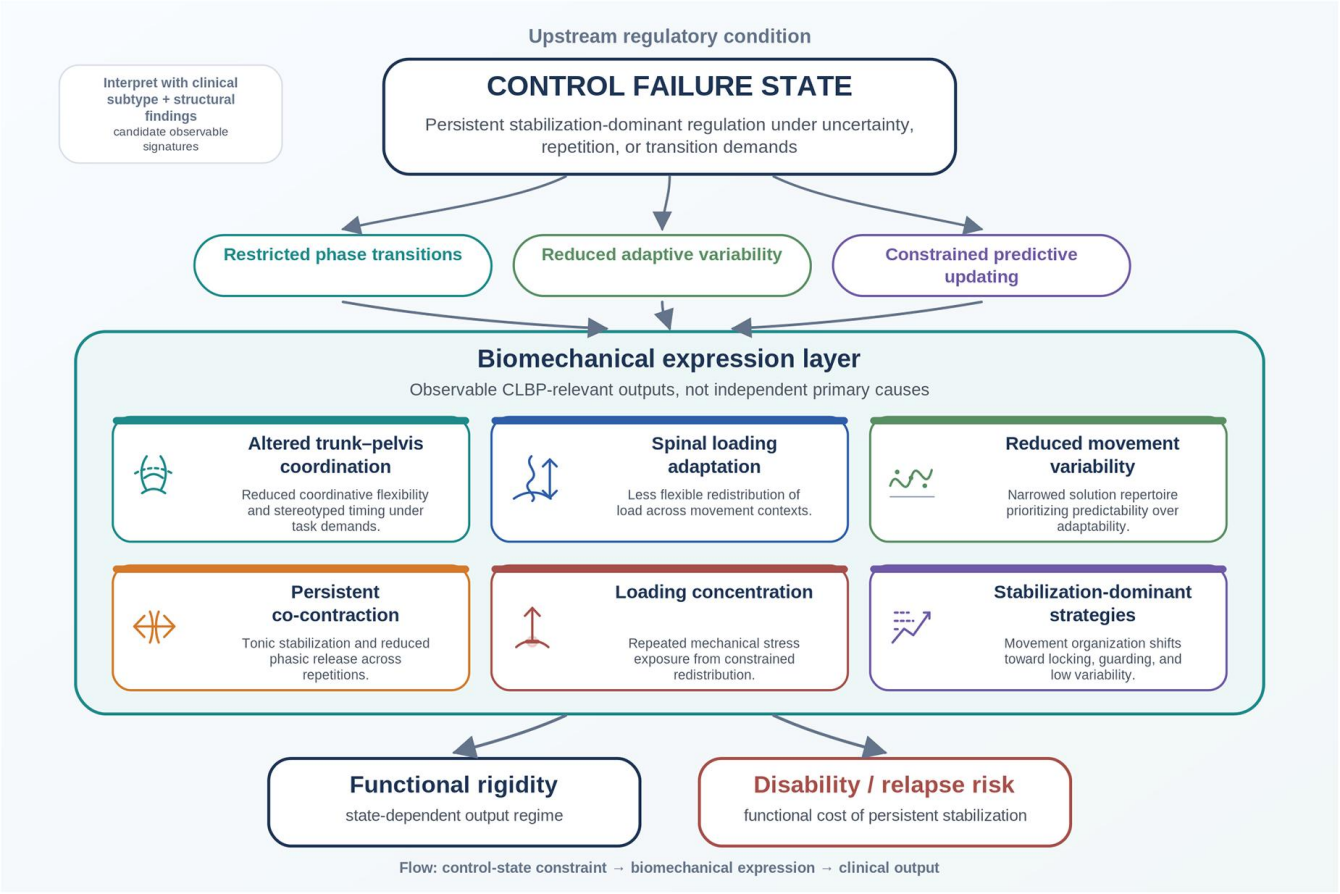
Biomechanical expression layer of control failure. The figure depicts the control failure state as an upstream regulatory condition that becomes clinically observable through altered trunk–pelvis coordination, spinal loading adaptation, reduced movement variability, persistent co-contraction, loading concentration, and stabilization-dominant movement strategies. These outputs are presented as biomechanical expression layers rather than independent causal mechanisms, culminating in functional rigidity and disability.

Before specifying FSCm outcome targets, the model's boundary conditions are summarized schematically in Figures 7, 8 specifically summarizes the biomechanical expression layer described in Section 3.5. Importantly, the proposed biomechanical expression layer represents a primary pathway through which a control failure state becomes clinically observable in CLBP. Trunk–pelvis coordination, loading redistribution, movement variability, and stabilization-dominant movement behavior are therefore treated as key observable manifestations of the underlying regulatory condition. The related scope and concept hierarchy are provided in Supplementary Text S0.

### 8.1 Why a constraint-level framework is necessary

Output-level correction can improve visible movement while leaving the underlying control state unchanged, particularly when threat-biased priors, reduced autonomic flexibility, and low tolerance for variability persist. A constraint-level account is therefore needed to explain persistence and relapse: what maintains the stabilization regime is the configuration of maintaining constraints that determine which control modes remain permissible. Here, constraints refer broadly to physiological, behavioral, and inferential factors that restrict phase transitions, variability modulation, and prediction updating.

### 8.2 The constraint landscape in CLBP: three maintaining classes

A more detailed taxonomy of maintaining constraints and the constraint landscape (including the three-class schema) is provided in Supplementary Text S1.

### 8.3 FSCm: four fixed outcome targets for modulating control failure constraints

FSCm specifies four constraint-level outcome targets that, in combination, characterize recovery from control failure. These targets are fixed at the level of mechanism (not intervention) and are intended to be operationalized through measurable marker changes, rather than to prescribe any specific treatment protocol.

#### 8.3.1 Phase transition restoration

A core target is re-opening the system's capacity to alternate regulatory phases through measurable phase-structured interfaces—such as respiratory phase alternation, trunk–pelvis or gait-related phase variability, and repeated-task movement markers—so that stabilization can be released and movement-based regulation can re-emerge.

#### 8.3.2 Adaptive variability expansion

A core target is restoration of variability as a functional control resource. This does not imply indiscriminate variability; it implies the return of flexible variability modulation—variability that can expand under uncertainty and contract when stability is required.

#### 8.3.3 Predictive updating re-enablement

A core target is re-establishing the system's capacity to revise predictions based on sensory evidence, reducing precision locking and allowing prediction error to drive learning rather than persistent stabilization.

#### 8.3.4 Multi-rhythm re-coupling

A core target is re-coupling across interacting rhythms and phase-structured interfaces (respiratory, autonomic, trunk–pelvis/gait-related, and motor/CPG-related rhythms) so that phase transitions and variability are coordinated rather than fragmented or rigidly locked.

Together, these outcome targets link the explanatory model (predictive–rhythm control failure) to modifiable targets and to measurable recovery signatures (phase alternation, autonomic flexibility, variability restoration, and improved performance under repetition). A schematic representation is provided in Supplementary Figure S1.

### 8.4 What changes first: a temporal ordering hypothesis

FSCm also yields a falsifiable temporal-ordering hypothesis: early recovery should be detectable first as improved phase-transition dynamics and autonomic flexibility, followed by re-expansion of motor variability and improved performance under repetitive demands.

In principle, clinical improvement can be decomposed into sequential signatures:

1. A selected phase-transition interface (e.g., respiratory phase alternation, trunk–pelvis/gait-related phase variability, or a repeated-task movement marker) becomes more flexible and less restricted →
2. Autonomic flexibility improves and becomes less locked (e.g., HRV or another autonomic index becomes more dynamically responsive) →
3. Motor rhythmic variability and stabilization output improve (reduced co-contraction; reduced phase fixation) →
4. Repetitive-task success and adaptability improve, potentially before pain fully resolves.

This ordering is posed as a falsifiable hypothesis rather than a universal rule; it can be tested by tracking lead–lag relationships across repeated measures. The specific phase-transition interface may vary by context, and respiration, trunk–pelvis coordination variability, gait-related phase variability, and repeated-task movement markers should be treated as complementary operational windows rather than competing causal explanations. The corresponding falsification criteria and temporal-order validation framework are summarized in Table 4.

**Table 4:** Falsifiable predictions and temporal-order validation framework for the predictive–rhythm control failure model. Lead–lag estimates are interpreted as testable ordering within standardized blocks and do not by themselves establish causality.

Prediction	Core claim (temporal ordering)	Primary markers	Methods/paradigms	Falsification criteria (what would disconfirm)
P1	Phase-transition interface marker improvement (e.g., respiratory phase alternation, trunk–pelvis/gait-related phase variability, or repeated-task phase marker) precedes or co-occurs with autonomic flexibility recovery.	Selected phase-transition interface indices (respiratory or biomechanical/repeated-task); ECG-derived HRV or other autonomic indices (standardized blocks)	Selected interface marker + ECG/autonomic index; baseline → transition → repetition blocks; standardize posture and measure/control breathing when respiratory metrics are used; estimate lead–lag (phase-locking/cross-correlation)	Autonomic flexibility improves reliably without any measurable change in the selected phase-transition interface across comparable task epochs, or ordering consistently reverses, or predicted coupling relationships are absent despite adequate measurement quality.
P2	Autonomic flexibility and phase-transition interface variability recovery precede reduction in stabilization outputs (co-contraction/tonic drive).	HRV or other autonomic indices + selected phase-transition interface; EMG co-contraction/tonic drive or IMU-based variability; repetition performance	ECG/autonomic index + selected interface marker + EMG/IMU during standardized repetition tasks (e.g., repeated sit-to-stand, gait, or lifting); quantify lead–lag and phase fixation; compare baseline and post-modulation blocks	Stabilization-based motor output (co-contraction/phase fixation) normalizes consistently before any preceding/co-occurring improvement in autonomic flexibility or selected phase-transition interface variability across comparable blocks.
P3	Variability recovery and repetition success can improve with minimal or non-proportional change in pain intensity (partial dissociation), and should explain variance in function beyond pain ratings.	Pain ratings + disability/function; repetition performance; variability/phase fixation markers; autonomic/interface indices	Multi-session tracking with standardized repetition probes; model variance explained beyond pain (mixed-effects) and assess mediation (pain vs. control markers); optionally estimate lead–lag across sessions	Variability and repetition performance improvements are consistently and fully explained by concurrent pain intensity change (pain fully mediates; no variance explained beyond pain), or control markers never improve unless pain decreases proportionally, contradicting partial dissociation claims.

A concise summary of the outcome targets and temporal-ordering hypothesis is provided in Supplementary Box S1.

### 8.5 Validation architecture and minimal measurement panel

FSCm is not proposed solely as a conceptual explanation of control failure. A central objective of the model is to generate an explicit validation architecture through which control failure can be operationalized, measured, and potentially falsified. The minimal panel prioritizes constructs that correspond directly to the three mechanistic features and four recovery targets: phase-transition capacity, autonomic flexibility, stabilization-related motor output, adaptive variability during repeated tasks, and functional disability.

Phase-transition capacity may be operationalized through multiple interfaces depending on context, including respiratory phase alternation, trunk–pelvis coordination variability, gait-related phase variability, and repetition-based movement markers. Respiration is emphasized primarily because of measurement feasibility and strong coupling with autonomic regulation, not because it is a required upstream pathway. The selected interface should be prespecified according to the dominant control failure signature and feasibility constraints.

**Figure 9:**
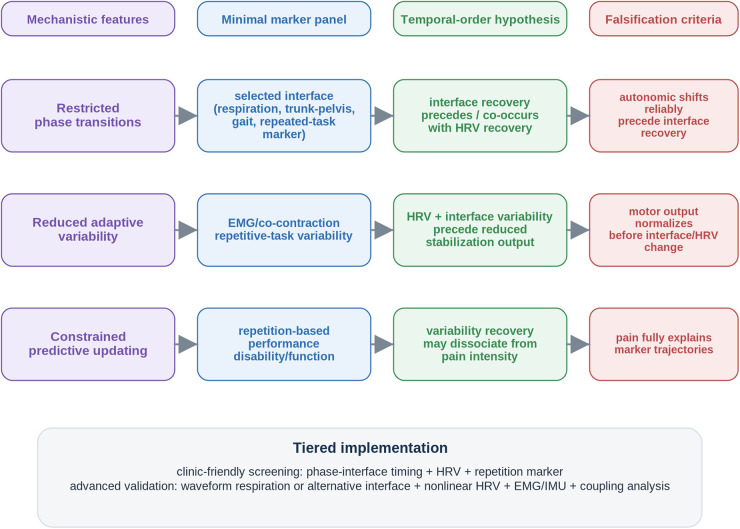
Validation architecture of FSCm. The figure maps the three mechanistic features (restricted phase transitions, reduced adaptive variability, constrained predictive updating) onto a minimal measurement panel (phase-transition interface, HRV, EMG/co-contraction, repetitive-task variability, disability), a temporal ordering hypothesis, and explicit falsification criteria. The term “phase-transition interface” is intentionally broader than respiration and may include biomechanical markers such as trunk–pelvis coordination variability, gait-related variability, or other repeated-task phase-transition measures when these provide more direct access to CLBP-related control dynamics.

This architecture is intentionally tiered. A pragmatic clinical validation tier may use symptom/function scales, simple phase timing, HRV, and repetition markers. A laboratory validation tier may add respiratory waveform analysis, EMG, inertial measurement unit (IMU)-based metrics or motion capture, and phase variability metrics. A mechanistic tier may add electroencephalography (EEG), neuroimaging, or cross-domain coupling analysis. The same temporal-order logic should be preserved across tiers. Importantly, the minimal validation panel was intentionally designed to remain feasible in routine clinical and rehabilitation settings using simple phase-transition timing measures, HRV assessment, repetition-based performance tasks, and basic motor variability markers, without requiring advanced neuroimaging or laboratory-based instrumentation. This validation architecture is summarized schematically in Figure 9, and the minimal validation panel is summarized in Table 5.

**Table 5:** Minimal validation panel and tiered validation architecture for FSCm.

Construct	Minimal marker	What it tests	Tiered extension
Phase-transition capacity	Respiratory phase timing, trunk–pelvis/gait-related phase variability, or another phase-structured interface	Whether the system can switch between stabilization and movement-generation states	Respiratory waveform, phase alternation metrics, transition timing, trunk–pelvis/gait phase variability, and other phase-structured interfaces
Autonomic flexibility	HRV under standardized rest/transition/repetition blocks	Whether autonomic state flexibility recovers with or before motor variability	Nonlinear HRV, respiration-adjusted HRV, autonomic coupling measures
Stabilization-related motor output	EMG co-contraction or IMU-based tonic/stability marker	Whether stabilization-dominant output decreases after phase/autonomic recovery	Multi-muscle EMG, tonic drive analysis, task-specific co-contraction indices
Adaptive variability under repetition	Repetition-task variability or phase fixation marker	Whether variability re-expands under uncertainty/repeated demand	Motion capture, trunk–pelvis variability, gait variability, phase-locking measures
Functional impact	ODI/RMDQ and repetition-task success	Whether control recovery explains function beyond pain intensity	Mixed-effects models, mediation/lead–lag analysis, longitudinal disability tracking

Core temporal hypothesis for validation: if CLBP motor dysfunction represents a persistent control failure state, recovery should follow a characteristic temporal sequence: phase-transition interface restoration → autonomic flexibility recovery → adaptive variability expansion → improved repetitive-task performance → pain reduction or disability improvement, with pain intensity allowed to change non-proportionally. This ordering is not treated as a universal rule but as a falsifiable lead–lag claim; if motor variability and stabilization output normalize consistently before any measurable improvement in phase-transition interface dynamics or autonomic flexibility, the proposed sequence would require revision.

## 9 Clinical assessment framework: measuring control failure rather than pain alone

A primary implication of the predictive–rhythm model is that symptom reduction and control restoration are separable. Pain intensity can decrease without resolving a stabilization-locked control regime, and range of motion can improve without re-establishing rhythmic adaptability under repetition or uncertainty. Therefore, assessment in CLBP should explicitly distinguish pain reduction from control recovery. This distinction is not a semantic preference; it is required to evaluate whether the proposed control failure state is present, whether constraint targets are being engaged, and whether recovery reflects a true return to movement-based regulation rather than compensatory performance in constrained tests.

### 9.1 Separating endpoints: pain reduction vs. control recovery

Pain is an important clinical outcome, but it is not a direct measure of phase-transition capacity, variability regulation, or prediction updating. Accordingly, assessment should distinguish symptom change from control recovery—especially under repetition and uncertainty. This distinction does not deny pain relevance; it predicts partial independence between symptom intensity and control-state restoration.

### 9.2 Repetition-based phase fixation as a system-level marker of control failure

Repetition-based phase fixation is proposed as a clinically feasible, system-level readout of stabilization-dominant control under repeated task demands. This marker class is intended as a proxy for repetition-linked phase fixation and is not intended to imply lumbar-specific causality. For CLBP-focused assessment, examples should prioritize reduced trunk–pelvis timing variability during repeated sit-to-stand, gait, or lifting blocks. Analogous non-lumbar repeated-task markers may be used when relevant, but CLBP-focused interpretation should prioritize trunk–pelvis timing, gait, sit-to-stand, and lifting performance. The interpretive value lies not in anatomy, but in whether phase transitions and adaptive variability can be restored across repetitions.

### 9.3 A panel approach aligned to constraint targets

Because FSCm is defined at the constraint level, assessment can be aligned to the four constraint-level outcome targets using a minimal marker panel. This panel and workflow are presented as research-oriented operationalization and hypothesis-testing tools; no clinical efficacy is claimed and implementation as routine care requires empirical validation. Candidate rhythm domains and broad marker classes are summarized in Table 2, whereas the minimal validation panel is summarized in Table 5 and expanded in Supplementary Table S1.

A minimal panel may include: (i) a phase-transition interface marker (respiratory phase alternation, trunk–pelvis/gait-related phase variability, or repeated-task phase marker), (ii) autonomic tone (HRV), (iii) motor rhythmic stability (EMG/kinematic variability), and (iv) repetition-based task performance. A more detailed assessment battery is provided in Supplementary Table S1. Practically, this can be organized as a two-level classification: Level 1 screening uses clinic-friendly snapshots (phase-interface timing + HRV+ a repetition marker), while Level 2 confirmation uses waveform respiration or another phase-interface analysis, nonlinear HRV, EMG/IMU, and coupling metrics. Because HRV is sensitive to confounds (e.g., breathing rate, posture, time-of-day, medication, and fitness), it should be interpreted under standardized conditions (or with respiration measured when respiratory metrics are used) and primarily via within-subject change across comparable blocks rather than absolute norms. If HRV is not feasible, the autonomic component of the panel can be tested with alternative autonomic indices while preserving the temporal-order logic. Evidence linking HRV to chronic pain has been summarized in systematic reviews (40, 41).

### 9.4 A clinical workflow for classifying signatures and selecting constraint targets

A mechanism-aligned workflow for classifying dominant signatures and selecting constraint targets is provided in Supplementary Figure S2.

## 10 Testable predictions and validation pathways

Hypothesis-and-theory models gain credibility when they generate a small set of specific, falsifiable predictions that can be tested with feasible methods. The present framework therefore concentrates on temporal ordering predictions: if control failure in CLBP is maintained by constraint-locked predictive–rhythmic desynchronization, then recovery should occur in an ordered manner as constraints are modulated. These predictions are intentionally limited in number to reduce ambiguity and to provide clear falsification criteria. The three core predictions and falsification criteria are summarized in Table 4.

A central principle guiding the predictions is that symptom change (pain intensity) is not equivalent to control recovery. The model therefore emphasizes markers that index phase transition capacity, autonomic flexibility, motor regime selection, and repetition-based adaptability, and it specifies when these markers should change relative to one another.

### 10.1 Core prediction set (three falsifiable claims)

P1. Phase-transition interface recovery precedes or co-occurs with autonomic flexibility recovery.

If phase-structured interfaces index transition capacity, then improvements in phase-transition interface markers should precede or co-occur with improvements in autonomic flexibility (e.g., HRV), particularly during task transitions or repetition blocks that demand phase switching. Minimal protocol: apply a repetition + transition task block while recording a selected phase-transition interface (respiratory waveform, trunk–pelvis/gait-related phase variability, or another repeated-task phase marker), electrocardiography (ECG)-derived HRV, and a simple motor output readout (EMG/IMU); test lead–lag using cross-correlation/phase-locking metrics; falsify if autonomic shifts reliably precede interface recovery across comparable task epochs.

P2. Autonomic flexibility and phase-transition variability recovery precede reductions in stabilization-based motor output.

If autonomic flexibility and phase-transition capacity gate variability and motor regime selection, then restoration of autonomic flexibility and phase-transition interface variability should precede measurable reductions in co-contraction and tonic drive, and should predict improved motor rhythmic stability under repetition. Minimal protocol: during repeated-demand blocks, quantify HRV, phase-transition variability (respiration or another measurable interface), co-contraction/tonic drive (EMG), and kinematic variability (IMU); falsify if motor output normalizes before autonomic/interface markers change.

P3. Variability recovery can partially dissociate from pain intensity change.

If control recovery reflects constraint modulation rather than simple symptom reporting, then improvements in motor variability (or phase fixation reduction) and repetition-based performance can occur with minimal or non-proportional changes in pain ratings and should explain variance in function/disability beyond pain intensity (e.g., mixed-effects models controlling for pain). Conversely, pain may improve while stabilization markers persist.

Additional exploratory predictions (e.g., repetition-based phase fixation and signature-specific responsiveness) are provided in Supplementary Text S2.

Minimal protocol (feasible 1-paragraph design): Use a within-session baseline → transition → repetition block while recording (i) a phase-transition interface marker (e.g., respiratory phase timing/waveform, trunk–pelvis or gait-related phase variability, or a repeated-task phase marker), (ii) ECG-derived autonomic indices (e.g., HRV, with respiration measured/controlled when respiratory metrics are used), and (iii) a simple motor output marker (IMU-based variability or EMG co-contraction/tonic drive). Estimate lead–lag relationships using phase-locking or cross-correlation across comparable epochs (phase-transition interface recovery → autonomic flexibility → reduced stabilization output → improved repetition performance). The temporal-order claims are falsified if ordering is reliably reversed under standardized blocks (e.g., stabilization markers normalize before interface/autonomic recovery) or if the predicted coupling relationships are absent despite adequate repetition stress.

### 10.2 Validation pathways: feasible study designs

These predictions can be tested using tiered validation pathways, ranging from pragmatic clinical protocols to controlled mechanistic studies. In particular, within-session and between-day variability should be treated as state trajectories: day-to-day fluctuations in function are expected to co-vary with selected phase-transition interface markers, autonomic flexibility, and motor variability markers if the regime-based account is correct.

#### 10.2.1 Within-session responsiveness (short-term ordering)

Measure phase-transition interfaces, autonomic flexibility, and motor variability during baseline, transition, and repetition blocks before and after targeted constraint modulation. Candidate interfaces may include respiratory phase alternation, trunk–pelvis coordination variability, gait-related variability, or other phase-structured markers. Test lead–lag relationships among phase-transition interfaces, autonomic flexibility, stabilization output, and adaptive variability.

#### 10.2.2 Multi-session clinical tracking (medium-term recovery)

Track marker trajectories across sessions to evaluate whether phase transition and autonomic changes precede changes in motor variability and repetition performance, and whether these changes predict disability improvement independent of pain.

#### 10.2.3 Signature-stratified trials (constraint-specificity)

Classify participants by dominant control failure signature (e.g., phase-transition failure present vs. absent; stabilization-dominant vs. non-stabilization-dominant pattern; biomechanical-expression-dominant vs. autonomic/respiratory-dominant profiles when relevant). Test whether interventions or exposures that target phase-transition restoration and variability expansion produce larger effects in signature-positive subgroups, and whether this moderation is mediated by early changes in phase-transition interface or autonomic markers.

#### 10.2.4 Mechanistic coupling analysis (advanced)

Quantify cross-domain coupling (e.g., interface–autonomic coupling, interface–movement phase coupling, respiration–HRV coupling when respiration is selected, or trunk–pelvis/gait phase coupling when biomechanical interfaces are selected) and test whether recovery corresponds to re-coupling rather than simple normalization of any single measure. This advanced tier is not required for pragmatic validation but can test the mechanistic claim that control recovery depends on restored flexible relationships among interacting rhythms.

### 10.3 Explicit falsification criteria and validation ladder

The model would be challenged if motor variability improves consistently before any measurable change in phase-transition interface dynamics or autonomic flexibility, if control failure signatures are absent despite persistent stabilization-dominant behavior, or if selected interface–autonomic changes consistently fail to precede motor adaptation across repeated observations.

Validation can proceed in three tiers. Tier 1 pragmatic clinical validation may use a minimal panel of phase-transition timing, HRV or another autonomic index, EMG/IMU-based motor output, and the Oswestry Disability Index (ODI) or Roland-Morris Disability Questionnaire (RMDQ). Tier 2 laboratory validation may add motion capture, repetition-task variability, and phase-transition metrics. Tier 3 mechanistic validation may add EEG, neuroimaging, and network-coupling analyses to test whether predictive, autonomic, and motor signatures become re-coupled during recovery.

## 11 Discussion and conclusion

FSCm should not be interpreted as a competing theory of CLBP. Instead, it is proposed as an integrative framework that connects predictive, behavioral, motor, biomechanical, and nociplastic processes through the concept of persistent control failure. The framework therefore focuses less on identifying isolated mechanisms and more on explaining how multiple disturbances become temporally stabilized and resistant to updating.

Nor should the framework be interpreted as a respiration-centered theory of CLBP. Respiration is used as one accessible indicator of phase-transition dynamics within a broader regulatory architecture. Future work may identify alternative physiological or behavioral markers with equal or greater explanatory value.

Similarly, FSCm should not be understood as a rejection of biomechanical explanations. Biomechanics is treated as an observable expression layer through which control failure becomes visible in movement behavior, spinal loading patterns, and the persistence of stabilization-dominant motor output.

### 11.1 Why does the movement pattern emerge?

Traditional accounts often attribute altered movement patterns in CLBP to pain avoidance, tissue protection, or learned compensation. The present model reframes this question at the control-architecture level.

Within the predictive–rhythm framework, altered movement patterns are not the primary defect but the observable expression of a stabilization-dominant control regime. When persistent perturbation biases precision allocation and narrows variability bandwidth, the system constrains phase transitions across coupled rhythms. As a result, anticipatory co-contraction and reduced variability emerge before movement initiation.

Thus, the movement pattern emerges not because movement is “wrong,” but because the control system has already shifted toward stabilization prior to action selection.

In this view, observable motor behavior is downstream of a time-ordered regulatory shift: precision bias → variability narrowing → phase-transition constraint → stabilization regime → movement expression.

If this ordering is incorrect, and movement deviation consistently precedes variability suppression or phase-transition changes, the model would require revision.

### 11.2 Why does correction fail?

Output correction strategies often improve visible kinematics or range of motion but fail to generalize across repetition, stress, or uncertainty.

Within the control failure model, this occurs because correction is applied at the output level while the constraint landscape remains unchanged. If phase transitions remain restricted and variability bandwidth remains narrow, the system reverts to stabilization under repeated demands.

Correction fails when:

precision locking persists,

autonomic inflexibility remains,

variability cannot re-expand under perturbation.

This explains why:

single-trial performance may improve,

but repetition reveals phase fixation,

and relapse risk remains elevated.

The model predicts that successful correction should involve measurable restoration of phase-transition capacity and autonomic flexibility prior to durable motor adaptability. Failure to observe this would falsify the constraint-level claim.

### 11.3 Why do stress and respiration modulate symptoms?

A central feature distinguishing this framework from conventional motor control impairment accounts is the integration of autonomic and respiratory phase dynamics into control architecture (42–44).

Stress increases arousal and biases precision allocation toward threat-weighted priors. This narrows variability tolerance and favors stabilization-based regulation. Because respiratory phase alternation is tightly coupled to autonomic flexibility, stress-induced reduced autonomic flexibility can constrain phase transitions.

Thus, symptom fluctuation under stress or altered breathing is not interpreted as psychological amplification alone, nor as isolated respiratory dysfunction. It reflects modulation of the same stabilization regime that governs motor output.

Respiration is positioned not as a privileged cause but as a clinically accessible interface through which phase-transition constraints become measurable.

If stress alters pain intensity without affecting respiratory variability, autonomic flexibility, or repetition-based stabilization markers, the coupling hypothesis would require refinement.

### 11.4 Why does performance fluctuate across days or contexts?

Perhaps the most clinically puzzling phenomenon in CLBP is variability across days:

“Why is movement possible today but not yesterday?”

Within this model, fluctuation reflects regime dominance rather than structural change.

When variability bandwidth and phase-transition capacity are partially restored, movement-based regulation can temporarily re-emerge. When perturbation, stress, fatigue, or contextual uncertainty increases, stabilization may again dominate.

Thus, performance fluctuation is not random inconsistency. It reflects dynamic competition between:

movement-based regulation

stabilization-based regulation

The degree to which stabilization persists across contexts defines the control failure state.

If day-to-day performance variation occurs independently of measurable changes in autonomic flexibility, respiratory alternation, or motor variability, the regime-based account would need revision.

### 11.5 Validation logic: why temporal ordering matters and how the model can fail

The model is most vulnerable—and therefore most useful—where it makes temporal claims about ordering (e.g., phase-transition dynamics → autonomic flexibility → motor variability). These falsifiable predictions, together with explicit disconfirmation criteria, are summarized in Table 4. Accordingly, the framework should be evaluated by its falsifiability and utility in guiding empirical design rather than by immediate empirical completeness.

The model could fail in interpretable ways. For example, if autonomic flexibility improves reliably without any change in the selected phase-transition interface across comparable task epochs, or if stabilization-based motor output normalizes before phase-transition/autonomic recovery, the proposed ordering would require revision.

- Stabilization-based motor output (co-contraction/phase fixation) normalizes consistently before any improvement in phase-transition interface dynamics or autonomic flexibility under standardized blocks.
- Repetition-induced phase fixation is absent under properly designed repetition stress tests (adequate intensity and duration) despite persistent disability/relapse risk, undermining the proposed persistence marker.
- Changes in variability and repetition performance are consistently and fully mediated by pain intensity change (no variance explained beyond pain), contradicting partial dissociation claims.
- Predicted coupling relationships (e.g., interface–autonomic coupling modulation; interface–motor phase alignment) are not observed despite adequate measurement quality and task demands.
- Alternative phase-structured interfaces (non-respiratory rhythms) provide a better or contradictory ordering, requiring revision of the proposed interface selection.

### 11.6 Boundary conditions and scope limitations

This manuscript is intentionally scoped to CLBP and to motor control impairment conceptualized as a control failure state. The framework does not claim that all CLBP presentations share a single signature, nor that respiration is the only possible interface. CLBP is used here as a focused instantiation because lumbar function depends strongly on stabilization–mobility switching and trunk–pelvis coordination under repeated loading (e.g., gait and lifting), and repetition-sensitive failure is clinically central. The model is most relevant when motor dysfunction, variability restriction, and relapse risk persist despite fluctuating pain intensity or apparent reductions in threat appraisal; it is less likely to apply in presentations dominated by acute structural instability, progressive neurological deficit, or conditions in which repetition does not expose phase fixation or transition failure.

The present framework also does not assume that all CLBP patients express control failure in the same manner. Some presentations may be predominantly stabilization-dominant, characterized by persistent co-contraction and reduced variability. Others may show partially preserved movement variability while retaining impaired phase-transition capacity or autonomic inflexibility. FSCm therefore emphasizes control failure signatures rather than fixed movement phenotypes, allowing different clinical presentations to converge on a shared regulatory framework while preserving clinical heterogeneity.

Clinical illustration of applicability: A patient with five years of recurrent CLBP, minimal structural findings, marked co-contraction during repeated sit-to-stand, reduced movement variability across repetitions, and strong symptom fluctuation despite repeated local correction would fit the proposed control failure signature. In such a case, FSCm would guide assessment toward phase-transition capacity, autonomic flexibility, stabilization output, and repetition-based variability rather than pain intensity alone.

Clinical illustration outside the primary scope: A patient with acute disc extrusion, severe radiculopathy, progressive neurological deficit, fracture, infection, or tumor-related pain should not be interpreted primarily through FSCm. In such presentations, structural, neurological, or red-flag management takes priority, although secondary control failure processes may be considered later if persistent motor dysfunction remains after appropriate management.

In addition, while the manuscript draws on a predictive regulation perspective, it does not attempt to adjudicate among all computational accounts of pain or motor control. The contribution is the specific integration of predictive regulation with multi-rhythm coordination and the constraint-level translation provided by FSCm. Further work should specify the computational formalization of constraints and precision locking, refine operational definitions for respiratory phase transition indices, and test cross-domain coupling metrics in longitudinal designs. Importantly, FSCm is presented as a constraint-level translation layer and does not propose a proprietary or specific intervention protocol. Additional hierarchy, clinical boundary examples, and biomechanical expression details are provided in Supplementary Texts S0, S3, and S4.

### 11.7 Implications for future research and implementation tiers

The framework suggests a pragmatic sequence for empirical work: establish reliable measurement of phase-transition interfaces, autonomic flexibility, and motor variability/co-contraction under standardized repetition paradigms, then test whether changes follow the predicted ordering and whether signatures stratify responsiveness.

Implementation can be tiered: a minimal clinic-friendly battery (e.g., symptom + phase-transition interface timing + HRV/autonomic snapshot + repetition-based task marker) can be used for screening and monitoring, while extended batteries (waveform respiration, nonlinear HRV, EMG, IMU-based kinematics, including trunk–pelvis/gait variability, and coupling analyses) can support mechanistic validation.

The present manuscript is intentionally positioned as a hypothesis-generating framework rather than a definitive explanatory model. Accordingly, the framework should be evaluated by its falsifiability and utility in guiding empirical design, rather than by immediate empirical completeness.

**Figure 10:**
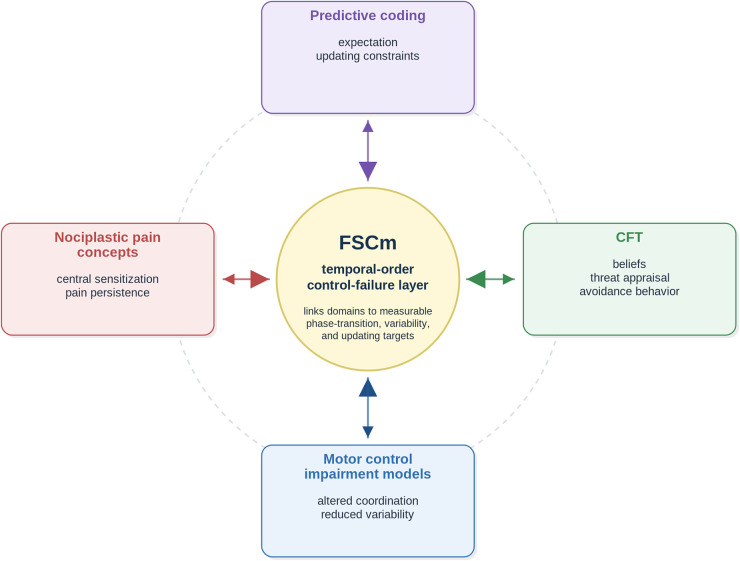
Relationship between existing CLBP theories and FSCm. Predictive coding, CFT, motor control impairment models, and nociplastic pain concepts are shown as complementary explanatory domains. FSCm does not replace these models; rather, it is positioned as an integrative layer explaining how these disturbances may become temporally coupled and stabilized within a persistent control failure state. The figure should be read as complementarity rather than replacement: predictive coding accounts for expectation and updating constraints, CFT emphasizes beliefs, threat appraisal, and avoidance behavior, motor control impairment models describe altered coordination and variability, and nociplastic pain concepts address central sensitization and pain persistence. FSCm adds a temporal-order control-failure layer linking these domains to measurable phase-transition, variability, and predictive-updating targets.

The complementary relationship between FSCm and existing CLBP theories is summarized in Figure 10.

### 11.8 Conclusion

Revisiting the four mechanistic questions clarifies that the core issue in CLBP motor dysfunction may not be movement error, but a failure of regulatory transition. This Hypothesis and Theory paper contributes a testable control-state construct for CLBP—defined by phase-transition capacity and repetition-induced phase fixation—and provides a constraint-level translation layer (FSCm) that yields explicit temporal-order predictions and a minimal measurement panel. FSCm is presented as a technique-agnostic translation layer that maps these claims to constraint-level outcome targets, not as a proprietary protocol or a clinical efficacy claim.

In CLBP, the proposed control failure state is expected to become clinically visible through altered trunk–pelvis coordination, reduced movement variability, loading concentration, and stabilization-dominant movement strategies. These biomechanical expressions do not replace predictive regulation as the explanatory level of the model; rather, they provide the observable pathway through which control failure becomes measurable in everyday movement behavior.

Within this framework, respiratory dynamics, trunk–pelvis coordination, gait-related variability, and other repeated-task phase-transition markers are viewed as complementary interfaces through which the control failure state may become measurable. The framework therefore remains focused on phase-transition dynamics themselves rather than on any single physiological or biomechanical marker.

The predictive–rhythm framework proposes that:

movement patterns emerge from stabilization dominance,

correction fails when constraints persist,

stress and physiological state modulate symptoms through phase-transition gating, with respiration treated as one accessible interface,

fluctuation reflects regime competition.

## Data Availability

The original contributions presented in the study are included in the article/Supplementary Material, further inquiries can be directed to the corresponding author.
