# Supplementary material for: Chronic low back pain as a control failure state: a predictive-rhythm model of motor control impairment and functional rigidity with functional system constraint modulation
Source: Front Pain Res (Lausanne). 2026 Jul 23;7:1812201. doi: 10.3389/fpain.2026.1812201 (PMC13442559; doi:10.3389/fpain.2026.1812201)
Supplement: Supplementary file 1 [file Table1.docx]

# Supplementary Text S0. Glossary, scope, hierarchy, and boundary conditions for FSCm

Reference numbers correspond to the reference list of the main manuscript.

## Supplementary Text S0.1. Glossary of core constructs and operational definitions

This glossary expands the brief definition block in the main manuscript. Each construct is defined at four levels: conceptual meaning, operational definition or candidate markers, clinical interpretation, and representative supporting literature. The glossary is intended to clarify terminology without adding new independent mechanisms.

**Control failure state.** Conceptual definition: A persistent stabilization-dominant regulatory regime in which the motor system can still produce movement but cannot readily restore flexible regulation once uncertainty, repetition, or perturbation is introduced. Operational definition/candidate markers: Operationally inferred when stabilization persists under repeated or transition-demanding tasks together with restricted phase-transition capacity and reduced adaptive variability. Pain intensity, structural pathology, or a single movement phenotype is not sufficient to define it. Clinical interpretation: Clinically, the patient may move, but movement remains narrow, guarded, repetitive, and poorly adaptable across contexts. Representative literature: (4,9,24).

**Predictive regulation.** Conceptual definition: Closed-loop regulation in which predictions about the sensory consequences of action are continuously compared with incoming evidence and revised to minimize expected future error under uncertainty. Operational definition/candidate markers: Assessed indirectly through the system’s capacity to update behavior when sensory evidence, task demands, or contextual constraints change. Clinical interpretation: Failure of predictive regulation is suggested when correction does not generalize and the system repeatedly returns to the same stabilization policy. Representative literature: (9,10).

**Predictive updating constraint.** Conceptual definition: A higher-order term for conditions in which prediction error no longer supports flexible learning and adaptation. Operational definition/candidate markers: Inferred from persistence of an outdated movement or threat model despite repeated evidence that alternative movement solutions are possible. Clinical interpretation: It explains why apparent correction can be transient when sensory evidence does not propagate into durable model revision. Representative literature: (9,10,35).

**Precision locking.** Conceptual definition: A stabilization-biased regulatory state in which threat-related priors are assigned excessive precision, reducing the capacity of incoming sensory evidence to update predictive models. Operational definition/candidate markers: Operationally inferred from convergent markers such as reduced adaptive variability, persistent stabilization-dominant motor behavior, impaired proprioceptive updating, and reduced autonomic flexibility. Clinical interpretation: Clinically, it may appear as co-contraction, repetitive movement strategies, and reduced exploration of alternative motor solutions. Representative literature: (9,10,12).

**Predictive-rhythmic desynchronization.** Conceptual definition: A loss of coordinated phase relationships among predictive, autonomic, respiratory, and sensorimotor rhythms. Operational definition/candidate markers: Operationally evaluated through convergent reductions in phase-transition capacity, adaptive variability, autonomic flexibility, and sensorimotor coordination. Representative markers may include decreased HRV, reduced trunk–pelvis coordination variability, increased movement stereotypy during repeated tasks, and impaired proprioceptive accuracy. Clinical interpretation: It is not proposed as a new physiological rhythm; it describes impaired coordination among existing regulatory rhythms that may contribute to functional rigidity, recurrent fluctuation, and impaired adaptability under repetition or transition demands. Representative literature: (7,18,30).

**Phase-transition interface.** Conceptual definition: Any measurable physiological or behavioral rhythm with explicit phase structure through which switching between stabilization and movement generation can be observed and perturbed. Operational definition/candidate markers: Respiration is one accessible example, but trunk-pelvis coordination, gait-related timing, repeated sit-to-stand dynamics, lifting organization, and autonomic rhythms may also serve as interfaces depending on the study context. Clinical interpretation: This term prevents respiration from being treated as a privileged cause and supports interface-general validation. Representative literature: (13,18,30).

**Restricted phase transitions.** Conceptual definition: A mechanistic feature of control failure in which the system has difficulty shifting between regulatory states such as stabilization, movement generation, recovery, and exploration. Operational definition/candidate markers: Operationally inferred from reduced phase alternation, delayed or incomplete task-to-rest or rest-to-task transitions, reduced autonomic switching, or persistence of phase fixation during repeated tasks. Clinical interpretation: Clinically, the patient may remain “held” or braced across contexts instead of smoothly entering and exiting movement states. Representative literature: (18,30,31).

**Adaptive variability / reduced adaptive variability.** Conceptual definition: Adaptive variability is functional variability that allows exploration, error correction, and context-dependent regulation. Reduced adaptive variability means that variability fails to expand when uncertainty or perturbation requires adaptation. Operational definition/candidate markers: Operationally assessed through kinematic variability, trunk-pelvis or gait variability, EMG variability, and repetition-based task performance under uncertainty or fatigue. Clinical interpretation: Clinically, reduced adaptive variability appears as a narrow repertoire of movement solutions and repeated return to the same stabilization strategy. Representative literature: (24,25,38).

**Functional rigidity.** Conceptual definition: The clinical or output-level expression of a control failure state. It refers to a state-dependent motor output regime rather than passive mechanical stiffness or simple ROM loss. Operational definition/candidate markers: Operationally characterized by anticipatory co-contraction, reduced phasic modulation, reduced rhythmic variability, impaired transition capacity, and repetition-sensitive phase fixation. Clinical interpretation: Clinically, functional rigidity explains why a patient may show temporary ROM or pain improvement while still failing to adapt during repeated or uncertain tasks. Representative literature: (4,5,24).

**Stabilization.** Conceptual definition: A regulatory strategy through which the system minimizes uncertainty by reducing variability, restricting phase transitions, and prioritizing predictability over adaptability. Operational definition/candidate markers: Operational markers include persistent co-contraction, elevated tonic drive, reduced adaptive variability, impaired phase-transition dynamics, reduced autonomic flexibility, and repetition-linked phase fixation. Clinical interpretation: In this framework, stabilization is not synonymous with desirable trunk stability. It can be useful acutely but maladaptive when it becomes the default regulatory policy. Representative literature: (4-6).

**Whole-body stabilization.** Conceptual definition: Extension of stabilization beyond the trunk into interacting motor, autonomic, respiratory, and behavioral systems. Operational definition/candidate markers: Operationally inferred when stabilization outputs appear across several domains rather than only at the painful region, for example co-contraction with reduced HRV flexibility and reduced phase-transition capacity. Clinical interpretation: Clinically, this construct captures the observation that CLBP-related motor control impairment may be expressed through whole-system regulatory narrowing rather than only local lumbar stiffness. Representative literature: (4,18,20).

**Avoidance-based stabilization.** Conceptual definition: A protective control strategy in which movement organization becomes biased toward minimizing anticipated threat rather than maximizing adaptability. Operational definition/candidate markers: Operationally inferred from persistent co-contraction, reduced movement variability, repetitive stabilization-dominant movement patterns, diminished exploratory motor behavior, and continued reliance on protective strategies despite reduced structural demand. Clinical interpretation: Clinically, it may appear as guarding, anticipatory muscle activation, repetitive movement solutions, and context-independent stabilization behavior. Representative literature: (5,6,35).

These constructs are not proposed as independently measurable entities. In FSCm, they are higher-order regulatory constructs inferred from converging patterns across autonomic flexibility, proprioceptive performance, movement variability, trunk–pelvis coordination, and repeated-task behavior. This operational approach is intended to support empirical testing while avoiding circular definitions.

**Constraint landscape.** Conceptual definition: The interacting physiological and behavioral constraints that determine whether adaptive variability, phase transitions, and predictive updating are permitted or suppressed. Operational definition/candidate markers: Operationally described through the relative contribution of constraints that block phase transitions, suppress adaptive variability, or prevent predictive updating. Clinical interpretation: Clinically, the constraint landscape explains why different CLBP presentations may show different dominant signatures while still belonging to the same control-failure subgroup. Representative literature: (24,30,33).

**Functional System Constraint modulation (FSCm).** Conceptual definition: A technique-agnostic, constraint-level validation architecture rather than a proprietary treatment protocol or universal CLBP theory. Operational definition/candidate markers: FSCm aligns three mechanistic features - restricted phase transitions, reduced adaptive variability, and constrained predictive updating - with four recovery targets: phase-transition restoration, adaptive variability expansion, predictive updating re-enablement, and multi-rhythm re-coupling. Clinical interpretation: Clinically and experimentally, FSCm specifies what should change first and which markers should be tracked to distinguish pain reduction from control recovery. Representative literature: (4,9,24).

**Multi-rhythm re-coupling.** Conceptual definition: Re-establishment of flexible coordination among phase-transition interfaces, autonomic rhythms, and sensorimotor/CPG-related motor rhythms. Operational definition/candidate markers: Operationally assessed by improved cross-domain coupling, such as respiration-HRV coupling when respiration is used, interface-movement phase coupling, HRV flexibility, and restored variability modulation during repeated tasks. Clinical interpretation: Clinically, re-coupling is expected to support recovery of movement-based regulation and reduce reliance on stabilization-dominant output. Representative literature: (7,18,42).

Functional System Constraint modulation (FSCm) is not intended to explain all presentations of chronic low back pain (CLBP). It is directed toward CLBP presentations characterized by a control failure signature: persistent stabilization-dominant motor behavior, reduced adaptive variability across repeated tasks, impaired phase-transition dynamics, disproportionate motor dysfunction relative to structural pathology, and limited symptom resolution despite conventional biomechanical correction.

The concept hierarchy used in the revised manuscript is as follows: core concept = control failure state; mechanistic features = restricted phase transitions, reduced adaptive variability, and constrained predictive updating; clinical expressions = functional rigidity, stabilization-dominant motor behavior, and motor control impairment. Precision locking is treated as one candidate form of constrained predictive updating; autonomic inflexibility and phase fixation are operational examples nested within these higher-order features rather than additional independent theories or separate competing constructs.

Respiration is treated as one practical phase-transition interface rather than as a privileged causal mechanism. In CLBP, phase-transition failure may also be observed through trunk–pelvis coordination, gait-related variability, repeated-task performance, and autonomic dynamics. The framework remains open to alternative physiological or behavioral interfaces if future research identifies markers that are more specific or more feasible in a given setting. Validation should therefore remain interface-general, selecting the phase-transition marker that best captures the relevant control dynamics for a given CLBP context rather than requiring respiration as a fixed upstream pathway.

Examples outside the primary explanatory scope include acute nociceptive pain, progressive neurological disorders, severe structural instability, tumor, infection, fracture-related pain, and other red-flag spinal conditions. These conditions require appropriate structural, neurological, or medical prioritization before FSCm-style control failure classification is considered.

## Supplementary Text S1. The constraint landscape in CLBP: three maintaining classes

For the target subgroup defined in Supplementary Text S0, we propose that the constraint landscape associated with maintaining control failure in CLBP can be described in three interacting classes:

1. Constraints that block phase transitions

Phase transitions enable the motor system to alternate between stabilization and movement generation. Respiratory phase alternation is treated here as an accessible example of a phase-transition interface rather than a privileged cause, and analogous phase-transition interfaces may be substituted depending on context. When phase alternation is restricted and autonomic flexibility is reduced, the system loses an important mechanism for switching regulatory states. This class of constraints includes persistent inspiratory bias, diminished expiratory relaxation, reduced respiratory variability, reduced autonomic state transitions, and impaired task-to-task switching between stabilization and movement generation.

2. Constraints that suppress adaptive variability

Adaptive variability is required for exploration, error correction, and context-dependent regulation. In control failure, variability is not simply reduced; it is inflexible, failing to expand when uncertainty or perturbation requires adaptation. This class includes persistent co-contraction, tonic drive elevation, reduced kinematic variability across repetitions, and a narrow repertoire of solutions (24-27,29,38).

3. Constraints that prevent predictive updating (precision locking as one candidate form)

Even when sensory evidence is available, prediction updating can remain constrained if precision allocation becomes rigid. Precision locking is treated here as one candidate form of constrained predictive updating: it limits learning from error and promotes stabilization persistence as a default strategy. This class also includes threat-weighted precision bias, reduced sensory sampling (which further diminishes informative evidence), and recurrent failure learning that reinforces conservative stabilization policies.

These classes interact. Phase transition restriction reduces autonomic flexibility and narrows state dynamics; reduced variability promotes fixation and reduces informative sensory sampling; precision locking further suppresses updating and encourages stabilization policies. The result is a self-reinforcing control failure state with a characteristic temporal signature: low rhythmic variability, impaired phase transitions, and stabilization outputs that persist across contexts.

Supplementary Box S1. FSCm outcome targets and temporal ordering (“what changes first”)

FSCm outcome targets (fixed; constraint-level, non-intervention-specific):

1. Phase transition restoration: target restoration of phase-transition capacity and autonomic state transitions that permit release from stabilization. Respiration is one accessible index, not a privileged causal mechanism.

2. Adaptive variability expansion: target re-expansion of functional variability and support restoration of flexible variability modulation across contexts.

3. Predictive updating re-enablement: target reduction of precision locking, treated as one candidate form of constrained predictive updating, and other updating constraints so prediction error supports learning and adaptation rather than persistent stabilization.

4. Multi-rhythm re-coupling: target re-establishment of coordinated coupling among phase-transition interfaces, autonomic rhythms, and motor/CPG rhythms to support stable phase transitions.

Temporal ordering hypothesis (what changes first):

Early recovery is expected to appear first as improved phase-transition dynamics (often captured pragmatically through respiratory phase alternation), followed by increased autonomic flexibility (e.g., HRV), then restored motor rhythmic variability (reduced co-contraction and phase fixation), culminating in improved repetitive-task success and adaptability, potentially preceding full normalization of pain intensity.

Validation tiers:
Tier 1 pragmatic: symptoms/function + simple phase timing + HRV + repetition marker.
Tier 2 laboratory: waveform respiration or alternative phase-interface analysis + EMG/IMU/motion variability.
Tier 3 mechanistic: EEG/neuroimaging/cross-domain coupling analyses.

## Supplementary Text S2. Additional exploratory predictions and extended validation options

P4. Normalization of trunk–pelvis or gait-related phase fixation reflects control recovery under demand.

If repetitive-task phase fixation is a marker of stabilization lock, then reductions in trunk–pelvis timing fixation during repeated sit-to-stand, gait, or lifting blocks should track control recovery, especially when assessed under repetition/uncertainty contexts, and should be more sensitive than single-trial kinematics or range of motion (ROM). Analogous non-lumbar repeated-task markers may serve as proxies for repetition-linked phase fixation when clinically relevant, but they are not intended to imply lumbar-specific causality.

P5. Signature-specific responsiveness: phase transition failure predicts larger constraint-modulation effects.

If targeted constraint modulation affects constraints that help maintain control failure, then individuals showing a dominant phase-transition failure signature (for example, restricted respiratory phase alternation, reduced flexibility in heart rate variability (HRV), or trunk–pelvis/gait-related phase fixation under repetition) should demonstrate larger effects from FSCm-aligned modulation targeting phase transitions and variability gating than individuals without this signature, indicating constraint-specific responsiveness.

## S2.1 Validation pathways: feasible study designs

These predictions can be tested using tiered validation pathways, ranging from pragmatic clinical protocols to controlled mechanistic studies:

## S2.1.1 Within-session responsiveness (short-term ordering):

Measure phase-transition interfaces, autonomic flexibility, and motor variability during baseline, transition, and repetition blocks before and after targeted constraint modulation. Candidate interfaces may include respiratory phase alternation, trunk–pelvis coordination variability, gait-related variability, or other phase-structured markers. Test lead–lag relationships among phase-transition interfaces, autonomic flexibility, stabilization output, and adaptive variability.

Schematic timeline (example): 2–3 min baseline rest → short transition probes (rest→task, task→rest) → 3–5 min repetition block (standardized cadence) → recovery rest. Estimate lead–lag within comparable epochs and treat ordering reversal under standardized blocks as falsification.

## S2.1.2 Multi-session clinical tracking (medium-term recovery):

Track marker trajectories across sessions to evaluate whether phase transition and autonomic changes precede changes in motor variability and repetition performance, and whether these changes predict disability improvement independent of pain.

## S2.1.3 Signature-stratified trials (constraint-specificity):

Classify participants by dominant signature (phase-transition failure present vs absent; biomechanical-expression-dominant vs autonomic/respiratory-dominant patterns). Test whether targeting phase-transition restoration yields larger effect sizes in the signature-positive subgroup and whether this moderation is mediated by early changes in phase-transition, autonomic, and repeated-task biomechanical markers.

## S2.1.4 Mechanistic coupling analysis (advanced):

Quantify cross-domain coupling (interface–autonomic coupling, such as respiration–HRV coupling when respiration is used; interface–movement phase coupling; trunk–pelvis/gait phase variability; variability modulation indices) and test whether recovery corresponds to re-coupling rather than simple normalization of any single measure (19,42-44).

Implementation notes:

“Phase-transition interface indices” may include inspiratory/expiratory timing variability when respiration is used, or trunk–pelvis/gait-related timing variability and other repeated-task phase markers when biomechanical interfaces are selected.

“Kinematic variability” should be evaluated under repetition/uncertainty contexts to reveal regime selection.

Falsification criteria are stated to preserve the theory’s vulnerability to disconfirmation.

# Supplementary Text S3. Clinical boundary examples and signature classification

FSCm-applicable example: persistent CLBP for several years, minimal or stable structural findings, recurrent symptom fluctuation, marked co-contraction during repeated movement, reduced repetition variability, and failure of symptom resolution despite repeated local correction. This pattern should be classified by dominant control failure signature and tested with the minimal panel before claims about control recovery are made.

Outside-primary-scope example: acute disc extrusion with severe radiculopathy, progressive weakness, fracture, infection, tumor-related pain, or severe structural instability. These presentations should be managed according to structural, neurological, or red-flag priorities. FSCm may only become relevant later if persistent stabilization-dominant motor dysfunction remains after appropriate management.

# Supplementary Text S4. Biomechanical expression layer

Biomechanics is treated as an observable expression layer rather than as a rejected or replaced explanatory domain. Control failure may become visible through altered trunk–pelvis coordination, spinal loading adaptation, reduced movement variability, persistent co-contraction, and stabilization-dominant movement strategies. These signs should be interpreted alongside structural findings and clinical subtype rather than used as stand-alone proof of control failure.

The proposed biomechanical expression layer therefore translates an upstream regulatory state into CLBP-relevant observable patterns. Together, these features provide one primary pathway through which control failure becomes measurable in everyday movement behavior. Altered trunk–pelvis coordination reflects reduced coordinative flexibility under changing task demands; spinal loading adaptation reflects reduced flexibility in how loading is distributed and redistributed across movement contexts; and reduced movement variability reflects a narrowed repertoire of movement solutions that prioritize stability over adaptability.

Because CLBP presentations are heterogeneous, the relative prominence of these biomechanical expressions may vary. Some presentations may primarily show stabilization-dominant trunk–pelvis coordination patterns and loading concentration, whereas others may demonstrate relatively preserved movement variability but persistent phase-transition constraints or autonomic inflexibility. The biomechanical expression layer should therefore be interpreted as a set of candidate observable control failure signatures rather than as a single mandatory biomechanical phenotype.

These biomechanical manifestations are not treated as independent causes of chronic low back pain. They are candidate measurable outputs of a stabilization-dominant regulatory regime and should be tested with repeated-task markers, trunk–pelvis or gait variability, loading-distribution measures, and co-contraction indices where feasible.

# Supplementary Figures

Supplementary Figure S1. FSCm as a constraint-level pathway from control failure to re-synchronization. The constraint landscape maintaining CLBP-related control failure is characterized by blocked phase transitions, suppressed adaptive variability, and predictive updating constraints that prevent flexible learning. FSCm is defined as a constraint-level translation layer that aligns these maintaining constraints with four outcome targets—phase transition restoration, adaptive variability expansion, predictive updating re-enablement, and multi-rhythm re-coupling—thereby enabling re-synchronization and recovery of movement-based regulation. Respiration is shown as one accessible interface rather than as a privileged driver.

Supplementary Figure S2. Mechanism-aligned assessment workflow for control failure in CLBP. The workflow separates symptom outcomes from control recovery by first screening target-population fit and red-flag exclusions, then assessing a targeted marker panel aligned with FSCm outcome targets (phase-transition interface, autonomic tone, motor rhythmic stability, and repetition-based performance). Patients are classified by dominant control failure signatures, candidate FSCm targets are considered accordingly, and recovery is verified through re-testing under repetition/uncertainty conditions to confirm restoration of phase transitions and adaptive variability.

# Supplementary Tables

Supplementary Table S1. Proposed assessment battery for distinguishing pain reduction from control recovery in CLBP.

| **Assessment domain** | **Minimal set (feasible clinic)** | **Extended set (research/advanced clinic)** | **What it tests (model link)** |
| --- | --- | --- | --- |
| Symptoms & function | Pain rating (e.g., numeric rating scale [NRS]), disability (e.g., Oswestry Disability Index [ODI]) | Daily variability diary; activity tolerance metrics | Symptom outcome vs control-state dissociation |
| Phase-transition interface | Respiratory phase timing or trunk–pelvis/gait-related phase marker; observation of transition flexibility under repetition | Respiratory waveform analysis; phase alternation indices; trunk–pelvis or gait phase variability; task-epoch transition timing metrics | Phase transition capacity (clinically accessible interface; FSCm outcome target 1; not limited to respiration) |
| Autonomic tone (HRV) | Resting + transition/repetition HRV epochs (within-subject change) | Rest + task-transition HRV; nonlinear HRV; respiration-adjusted HRV coupling | State flexibility / autonomic gating (outcome targets 1-2) |
| Motor rhythmic stability | Simple repetition test with timing/consistency score | EMG co-contraction indices; kinematic variability; phase-locking metrics | Stabilization regime vs adaptive variability (outcome targets 2-4) |
| Representative phase fixation marker | Repeated sit-to-stand, gait, or lifting repetition marker with trunk–pelvis timing/consistency observation | Trunk–pelvis or gait phase fixation quantified via IMU/kinematics ± EMG; analogous non-lumbar phase-fixation marker only when relevant | Persistent control failure signature under CLBP-relevant repetition demand (system-level proxy; not a site-specific causal claim) |
| Repetitive task performance | Repetition success rate; fatigue-related decline | Repetition blocks with variability metrics; perturbation paradigms | Control recovery independent of pain (exploratory prediction) |
| Cross-domain coupling | — (optional) | Interface–autonomic coupling; respiration–HRV coupling when respiration is used; interface–movement phase coupling; trunk–pelvis/gait phase coupling | Multi-rhythm re-coupling (outcome target 4) |
| Scope / boundary screening | Red-flag screen; structural-pathology dominance; control failure signature checklist | Clinical subtype stratification; imaging/neurological correlation when indicated | Determines whether FSCm is applicable as a primary explanatory framework |
| Biomechanical expression layer | Task observation for trunk–pelvis coordination, spinal loading adaptation / loading concentration, co-contraction, reduced movement variability, and repeated-task decline | Trunk–pelvis kinematics, gait variability, load-distribution analysis, EMG co-contraction, repeated sit-to-stand/lifting variability metrics | Tests whether control failure becomes visible as persistent stabilization-dominant biomechanical expression rather than as an independent primary cause |
| Existing-model linkage | CFT-relevant behavior/threat screen; motor control phenotype screen | Prediction-error tasks; nociplastic pain markers; combined behavioral-motor profiling | Tests whether FSCm adds integrative value beyond isolated explanatory domains |

Abbreviations: CFT, Cognitive Functional Therapy; CLBP, chronic low back pain; CPG, central pattern generator; ECG, electrocardiography; EEG, electroencephalography; EMG, electromyography; FSCm, Functional System Constraint modulation; HRV, heart rate variability; IMU, inertial measurement unit; NRS, numeric rating scale; ODI, Oswestry Disability Index; ROM, range of motion.
